# Structural insights into the activation and inhibition of the ADAM17–iRhom2 complex

**DOI:** 10.1073/pnas.2500732122

**Published:** 2025-06-13

**Authors:** Joseph J. Maciag, Conner E. Slone, Hala F. Alnajjar, Maria F. Rich, Bryce Guion, Igal Ifergan, Carl P. Blobel, Tom C. M. Seegar

**Affiliations:** ^a^Department of Molecular and Cellular Biosciences, University of Cincinnati College of Medicine, Cincinnati, OH 45267; ^b^Departments of Medicine and of Biochemistry, Cell and Molecular Biology, Weill Cornell Medicine, New York, NY 10021; ^c^Arthritis and Tissue Degeneration Program, Hospital for Special Surgery, New York, NY 10021

**Keywords:** ADAM17, iRhom2, ectodomain shedding, immunity

## Abstract

The endopeptidase activity of ADAM (a disintegrin and metalloproteinase)-17, the primary processor of several EGFR ligands and tumor necrosis factor-alpha (TNF-α), is essential for proper embryonic development and immune regulation. Dysregulated ADAM17 activity is prevalent in a wide array of human diseases, including cancer, chronic inflammation, and SARS-CoV-2 viral progression. Initially translated as an inactive zymogen, ADAM17 maturation and enzymatic function are tightly regulated by its obligate binding partners, the inactive rhomboid proteins (iRhom) -1 and -2. Here, we present the cryo-EM structure of the ADAM17 zymogen bound to iRhom2. Our findings elucidate the interactions within the ADAM17–iRhom2 complex, the inhibitory mechanisms of the therapeutic MEDI3622 antibody and ADAM17 prodomain, and the previously unknown role of a membrane-proximal cytoplasmic reentry loop of iRhom2 involved in the mechanism of activation. Importantly, we perform cellular assays to validate our structural findings and provide further insights into the functional implications of these interactions, paving the way for developing therapeutic strategies targeting this biomedically critical enzyme complex.

## Significance

ADAM (a disintegrin and metalloproteinase)-17 is a crucial enzyme that activates proteins involved in development, immune defense, and tissue repair. Its dysregulation is linked to diseases such as cancer, autoimmune conditions, and COVID-19. Using advanced cryogenic electron microscopy (cryo-EM) techniques, we resolved the structure of ADAM17 bound to its regulator protein, inactive rhomboid proteins (iRhom)-2, and identified key structural elements essential for its activity and regulation. We uncovered a key regulatory element within the cytoplasmic domain iRhom2 and clarified the role of the ADAM17 prodomain, challenging prior activation models. Additionally, we detailed the inhibitory mechanism of the therapeutic antibody MEDI3622. This work provides a foundation for designing therapies targeting ADAM17-related diseases, offering strategies to address critical human health conditions.

Ectodomain shedding is a biological process whereby proteolytic release of membrane-tethered proteins allows cells to communicate with one another and interpret their extracellular environment (1). The ADAMs (a disintegrin and metalloproteinase) are type-1 transmembrane proteins with endopeptidase activity that participate in ectodomain shedding. ADAMs are widely expressed throughout the body with essential functions in fertilization, cell differentiation, angiogenesis, immunity, and development of nervous and epithelial tissues (2, 3). Correspondingly, dysfunctional ADAM activity is thought to contribute to a variety of pathological conditions; such as autoimmune diseases, cancer, and Alzheimer’s disease (1, 4–7).

Among the ADAM family, ADAM17 stands out for its essential roles in epidermal growth factor receptor (EGFR) signaling during embryonic development, adult homeostasis, and disease pathogenesis (2, 8–10). ADAM17 was originally identified as the enzyme responsible for the release of soluble tumor necrosis factor-α (TNFα), a potent inflammatory cytokine, from cells (11, 12). Genetic ablation of ADAM17 in mice results in perinatal lethality, with mutant mice displaying open eyes at birth and defects in heart valve and growth plate formation, which has been attributed to a loss in EGFR signaling (8, 9, 13–15). Patients lacking ADAM17 have severe skin and intestinal barrier defects (16, 17), likely caused by defects in the activation of EGFR-ligands (18, 19). Accordingly, ADAM17 was identified as the enzyme responsible for the maturation of several pro-EGF ligands into soluble signaling molecules (8, 9, 10, 20, 21). In addition to its function as the TNFα convertase, ADAM17 also cleaves a myriad of other membrane tethered proteins to shed them from the cell membranes (3), including the interleukin 6 receptor (IL-6R) (5, 8, 13, 22, 23), a key player in autoimmune disease.

During biosynthesis, ADAM17 is translated in the endoplasmic reticulum (ER) as a zymogen, with a prodomain tightly bound to its catalytic domain to maintain enzyme latency and prevent premature substrate processing (24). Maturation of ADAM17 from its zymogen form, a prerequisite for peptidase activity, involves the proteolytic processing of the prodomain by proprotein convertases in the trans-Golgi network (24, 25). The mature ADAM17 contains an extracellular metalloproteinase (M), ancillary disintegrin (D) and cysteine-rich (C) domain, a single transmembrane (TM) α-helix, and a cytoplasmic domain (SI Appendix, Fig. S1*A*) (11, 12).

ADAM17 activity can be rapidly stimulated, with substrate shedding induced within minutes of activation (20, 26–28). The short temporal activation and inability to increase enzyme activity by systemic overexpression in mice (29) highlight the posttranslational nature of the mechanism governing ADAM17 activation. While the ADAM17 cytoplasmic domain is required for normal mouse development (30), paradoxically, stimulated ADAM17-dependent shedding occurs independent of its cytoplasmic domain (26, 31) but requires the presence of its transmembrane domain (28). These observations underscore the importance of one or more other integral membrane proteins in regulating ADAM17 activity. To this end, the discovery of the seven-transmembrane inactive rhomboid proteins (iRhom1 and iRhom2) as essential regulators of ADAM17 revealed the identity of its crucial regulatory binding partners (32–35).

iRhoms are part of the rhomboid protease superfamily of proteins that have evolved to include a large extracellular domain (ECD) and have lost the amino acids required to function as an integral membrane protease, making them functionally distinct from other rhomboid protease family members (36–38). Mice lacking iRhom2 fail to generate mature and functional ADAM17 in hematopoietic lineage cells, which therefore release little, if any, soluble TNFα from stimulated immune cells in proinflammatory conditions (32, 33). Yet, unlike *Adam17^−/−^* mice, which die shortly after birth, mice lacking iRhom2 have no spontaneous pathological phenotypes (5, 32, 33). However, double knockout mice lacking *iRhom1* and *iRhom2* die shortly after birth with open eyes, heart valve, and growth plate defects, thus closely resembling mice lacking *Adam17* (35). No mature, functional ADAM17 can be detected in *iRhom1/2^−/−^* cells and tissues, including in a second *iRhom1/2-*deficient mouse strain that dies during embryogenesis (34). These findings highlight the essential role of iRhoms in the maturation and function of ADAM17. Moreover, these studies suggest that either iRhom1 or iRhom2, which share 61% sequence identity, can bind ADAM17 zymogen to facilitate its exit from the ER and permit ADAM17 maturation in the trans-Golgi network (34, 35). iRhoms are also thought to accompany ADAM17 *en route* to the cell surface, where they regulate its extracellular peptidase activity (39–44). A point mutation identified in the TM1 of iRhom2 in mice, termed *sinecure*, strongly reduces the maturation and function of ADAM17, establishing the importance of the iRhom2 TM1 as an essential component in ADAM17 regulation (45, 46). Replacing the TM of ADAM17 with that of betacellulin gives rise to a constitutively active protease that can no longer be rapidly stimulated and does not require the iRhoms for its catalytic activity (28, 47). Moreover, cells lacking ADAM17 have little, if any iRhom2 (48), whereas immune cells lacking iRhom2 have no mature ADAM17 (32, 33), establishing a reciprocal dependence of both binding partners for their maturation and function. Thus, iRhoms are considered vital regulators of ADAM17 activity.

Here, we report a high-resolution cryo-EM structure of the ADAM17 zymogen bound to iRhom2 and the MEDI3622 therapeutic antibody and the results of structurally informed cell-based assays to identify key features of the complex essential for ADAM17 activity. Critically, beyond reinforcing recent structural work (49), we identify a membrane-proximal cytoplasmic region in iRhom2 that integrates into its transmembrane α-helix bundle and appears to be crucial for stimulation of ADAM17 sheddase function. Additionally, we provide atomic insights into the inhibitory mechanisms of the MEDI3622 therapeutic antibody and the ADAM17 prodomain in blocking ADAM17 catalytic activity. These findings deepen our understanding of ADAM17 regulation and function and will inform the development of therapeutic strategies targeting this key enzyme complex.

## Results

### Structure of the Zymogen ADAM17–iRhom2 Complex.

To establish optimal expression and isolation procedures for structural studies, we first created a series of iRhom2 cytoplasmic deletion constructs, replacing portions of this domain with the fluorescent reporter protein mVenus (SI Appendix, Fig. S1*A*). The iRhom2 cytoplasmic domain is not predicted to bind the ADAM17 cytoplasmic domain, a region dispensable for stimulated endopeptidase activity (26, 28, 50). The chimeric mVenus–iRhom2 proteins, along with ADAM17, were expressed in Expi293 cells, detergent solubilized, and screened using fluorescent-based size exclusion chromatography (FSEC) to identify optimal conditions to isolate the ADAM17–iRhom2 complex (SI Appendix, Fig. S1 *B* and C). Interestingly, iRhom2 mutants with truncated forms of the cytoplasmic domain were less susceptible to sample degradation than full-length iRhom2 and still retained the ability to bind ADAM17.

**Fig. 1.**
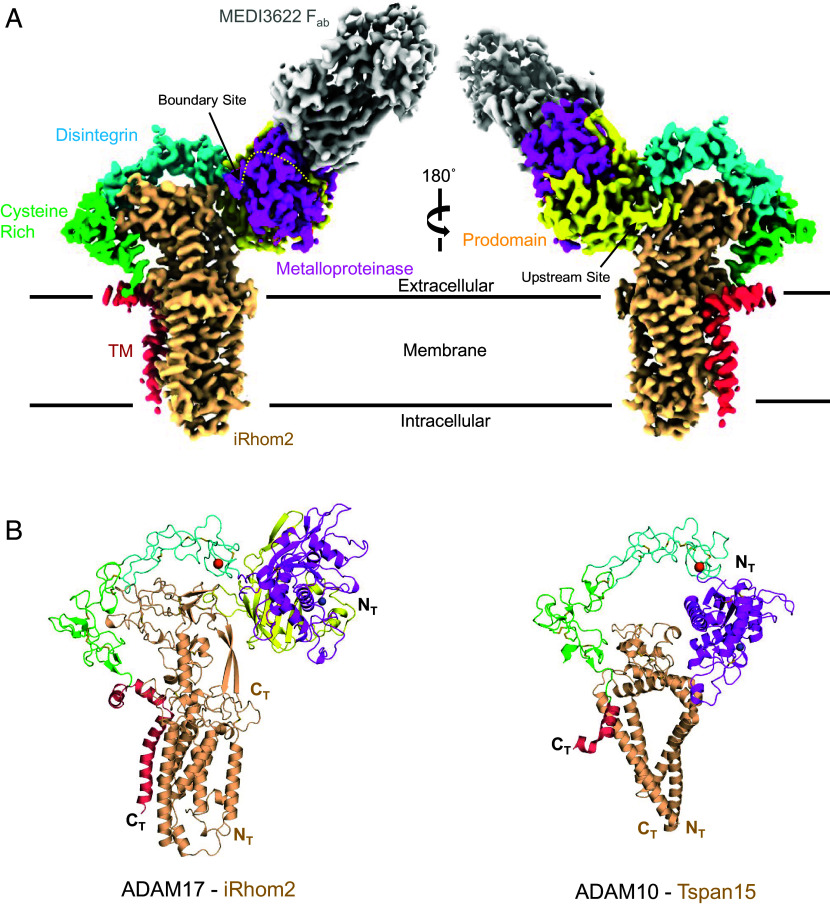
Overall structure of the iRhom2–ADAM17 zymogen complex. (*A*) Cryo-EM density map of the zymogen ADAM17–iRhom2–MEDI3622 F_ab_ complex embedded within the cell membrane, represented by parallel black lines. ADAM17 is colored according to its structural domains: Prodomain (yellow), Metalloproteinase (magenta), Disintegrin (cyan), Cysteine-Rich (green), and TM domains (red). iRhom2 is shown in tan, and MEDI3622 F_ab_ is depicted in gray. Proprotein convertase cleavage sites within the prodomain are indicated by arrows, denoting the Upstream (US) and Boundary (BS) cleavage sites. (*B*) Cartoon representation comparing the zymogen ADAM17–iRhom2 complex (*Left*) and the ADAM10–TSPAN15 complex (*Right*, RCSB PDB: 8ESV). ADAM17 and ADAM10 structures were superimposed based on their D + C domains and then separated for visualization. Structural domains are color-coded as in (*A*), with additional representation of calcium ions (orange) and catalytic zinc ions (dark gray) as spheres. The cytoplasmic domains for both ADAM17 and iRhom2 were not resolved in the final map and are not depicted.

To determine the structure of the complex, the mVenus-Δ363-iRhom2 cytoplasmic deletion construct and ADAM17 with the inactivating protease mutation E406A were coexpressed in Expi293 cells. The complex was solubilized in detergent buffer and purified using an αGFP-nanobody affinity column. The purified zymogen ADAM17–iRhom2 complex was then bound by a F_ab_ fragment, corresponding to a function blocking α-ADAM17 antibody (MEDI3622) (51), to learn more about its mechanism of inhibition and to serve as a fiducial marker for cryo-EM imaging and structure determination (SI Appendix, Fig. S1*D*). The structure of the zymogen ADAM17-MEDI3622 F_ab_ complex bound to iRhom2 was determined using single particle analysis to a global resolution of 3.53 Å (Fig. 1 and SI Appendix, Fig. S2). The final density map displayed clear structural features for zymogen ADAM17 and iRhom2 embedded in a glycol-diosgenin (GDN) micelle, with the MEDI3622 F_ab_ bound to the ADAM17 ECD. However, no density was observed for the cytoplasmic regions of ADAM17 or the iRhom2-mVenus fusion, likely due to native disorder and high flexibility in the cytoplasmic domains. To build the initial structure, AlphaFold predictive models for the MEDI3622 F_ab_, Δ363-iRhom2 cytoplasmic deletion mutant and individual domains of ADAM17 were docked into the final density map, manually rebuilt, and refined (Table 1). Manual inspection of the final protein model in the density map showed good correlation between the Cα-backbone and placement of large branched amino acid side chains into density extending from the secondary structural elements.

**Table 1.** Cryo-EM data collection and refinement statistics

	F_ab_-A17-iRhom2	F_ab_-A17(PM)
**Data collection and processing**	PDB: 9O58	PDB: 9O54
Magnification	105 kX	165 kX
Voltage (kV)	300	200
Total dose (e^−^/Å^2^)	62.2/56.30	40
Defocus range (µm)	0.4 to 2.2	0.4 to 2.0
Pixel size (Å)	0.822	0.69
Symmetry	C1	C1
Initial particle images	2,797,927	2,816,334
Final particle images	46,753	98,475
Map resolution (Å)	3.53	3.5
Map resolution range (Å)	1.93 to 44.85	2.11 to 40.27
FSC threshold	0.143	0.143
**Refinement**		
Model resolution (Å)	3.97	3.80
FSC threshold	0.5	0.5
Model composition		
Non-hydrogen atoms	12,422	6,432
Protein residues	1,567	827
Ligands	10	1
B-factors (Å^2^)		
Protein	122.41	80.25
Ligands	138.18	76.04
RMS devations		
Bond lenghts (Å)	0.004	0.008
Bond angles (∘)	0.654	1.018
Validation		
MolProbity score	2.00	2.00
Clashscore	13.31	13.17
Rotamer outliers (%)	0.36	0.42
Ramachandran plot		
Favored (%)	94.73	94.61
Allowed (%)	5.27	5.39
Disallowed (%)	0	0

The overall architecture of the zymogen ADAM17–iRhom2 complex reveals an extended conformation of the ADAM17 ECD, positioned proximal to the cell membrane making extensive contact with the iRhom2 ECD, known as the iRhom homology domain (IHD) (Fig. 1). Both proteins are further stabilized and anchored in the membrane by their TM α-helices. The inhibitory MEDI3622 F_ab_ projects from the ECDs, bound to the ADAM17 metalloproteinase (M) domain. Remarkably, the overall structure of zymogen ADAM17 bound to iRhom2 resembles the conformation adopted by ADAM17’s closest homolog, ADAM10, bound to the multipass transmembrane tetraspanin 15 (Tspan15) protein (RCSB PDB: 8ESV) (52). Superimposition of the ADAM17 ancillary D + C domains onto ADAM10 (RMSD = 2.65 Å) positions the transmembrane binding partners, Tspan15 and iRhom2, as a molecular bridge between the ADAM C and M domains (Fig. 1*B*). Unlike the ADAM10–Tspan15 structure, the ADAM17–iRhom2 complex shares extensive contact within the transmembrane regions.

### Inhibition of ADAM17.

**Fig. 2.**
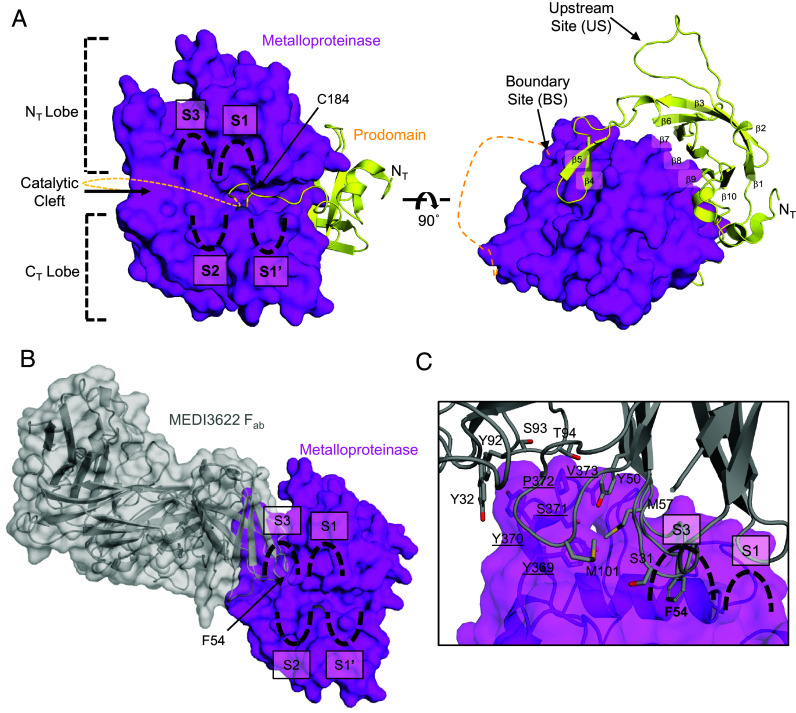
Structural analysis for the inhibition of ADAM17. (*A*) Cartoon representation of the prodomain and metalloproteinase domain of ADAM17, with the metalloproteinase domain surface shown in transparency. The metalloproteinase domain is divided into two lobes, N_T_ and C_T_ (dashed brackets), based on their positions relative to the central catalytic cleft containing the substrate-selective S3, S2, S1, and S1′ pockets (outlined). Structural β-strands (β1–10) of the prodomain and the cysteine switch residue, C184 (side chain depicted as sticks with RGB coloring), are annotated. The C_T_ region of the prodomain, including the BS cleavage sites, is represented as a dashed line. (*B*) Cartoon and surface representation of the MEDI3622 F_ab_ (gray) bound to the ADAM17 metalloproteinase domain, highlighting F54 of the MEDI3622 F_ab_, which occupies the S3 substrate-selective binding pocket. (*C*) Zoomed-in view of the amino acids within the contact interface between the MEDI3622 F_ab_ and the ADAM17 metalloproteinase domain (underlined).

The ADAM17 M domain is divided into N-terminal (N_T_) and C-terminal (C_T_) lobes (Fig. 2*A*), separated by a central catalytic cleft that uses enzyme pockets, denoted as S, to position an extended protein substrate for proteolysis. The enzyme S pockets are numbered relative to the scissile bond location, with positions toward the N-terminal given unprimed notation (e.g., S1, S2), and those toward the C-terminal given primed notation (e.g., S1′, S2′). The active site of ADAM17 harbors a zinc ion coordinated by three histidine residues, along with a glutamic acid residue and a water molecule, which together facilitate the hydrolysis of a protein substrate between the S1 and S1′ recognition pockets (53). In addition to our zymogen ADAM17–iRhom2 structure, we determined the structure of the MEDI3611 F_ab_ bound to the ADAM17 Pro-M domains, which readily superimposes with the zymogen structure (RMSD = 1.31 Å) (Table 1 and SI Appendix, Fig. S3). In our structures, the prodomain is observed bound to the ADAM17 M domain and wedged between this domain and the iRhom2 IHD (Figs. 1 and 2). Unlike other proteinase prodomains, which are typically short polypeptides, the ADAM17 prodomain is considerably larger, approximately 25 kDa, with no homologous structure available in the RCSB database. The ADAM17 prodomain folds into two stacked β-sheets, forming a β-barrel motif (β1-3 and β5-10) that binds to the M domain opposite the active site cleft, burying a substantial contact interface of 5,380 Å^2^ (Fig. 2*A*). Additional noncatalytic cleft contacts of the prodomain extend the central β-sheet in the M domain by two β-strands (β3 and β4). The C-terminal end of the prodomain, referred to as the “cysteine-switch,” contains a single cysteine residue (C184) whose thiol side chain coordinates the active site zinc ion. Though the C184 residue itself is dispensable for inhibition in cellular assays and in studies with recombinant ADAM prodomain (54, 55), the region preceding C184 also occupies the catalytic cleft and is critical for inhibiting ADAM17 activity (Fig. 2). To summarize, prodomain interactions inhibit ADAM17 by displacing the nucleophilic water molecule and sterically blocking substrate access.

Additionally, our structures elucidate the precise molecular mechanism of inhibition by the therapeutic antibody MEDI3622, demonstrating how its binding to the ADAM17 M domain obstructs substrate access and prevents proteolytic activity (Fig. 2 *B* and *C*). The complementarity-determining regions (CDRs) of the MEDI3622 F_ab_ binds to a loop, distal from the active site, in the ADAM17 M domain, specifically at amino acids Y369/Y370/S371, confirming the binding epitope previously identified by mutagenesis (Fig. 2 *B* and *C*) (56). Substrate selectivity studies for ADAM10 and ADAM17 indicate a preference for amino acids up to four residues from the scissile bond, particularly favoring branched amino acids that would occupy the S3 selective pocket (57, 58). In support of this, the X-ray structure of the ADAM10 ECD shows a peptide product occupying the S1–S3 substrate-selective pockets (59). Notably, the placement of the F54 side chain in the CDR2 of the MEDI3622 F_ab_ heavy chain into the ADAM17 hydrophobic S3 substrate-selective pocket sterically obstructs an incoming protein substrate, which is crucial for ADAM17 activity. Moreover, the binding of MEDI3622 likely accounts for the absences of density for the prodomain region between the cysteine-switch and the proprotein convertase processing site, which resides at the boundary between the pro- and M domains in our structures. This region likely binds to the S3 pocket, as these amino acids have been implicated in the inhibitory properties of the ADAM17 prodomain (54). Altogether, the zymogen ADAM17-MEDI3622 cryo-EM structures provide atomic-level details of how MEDI3622 inhibits ADAM17 activity, effectively preventing access of the substrate to the catalytic cleft.

### Overall Structure of the iRhom2.

**Fig. 3.**
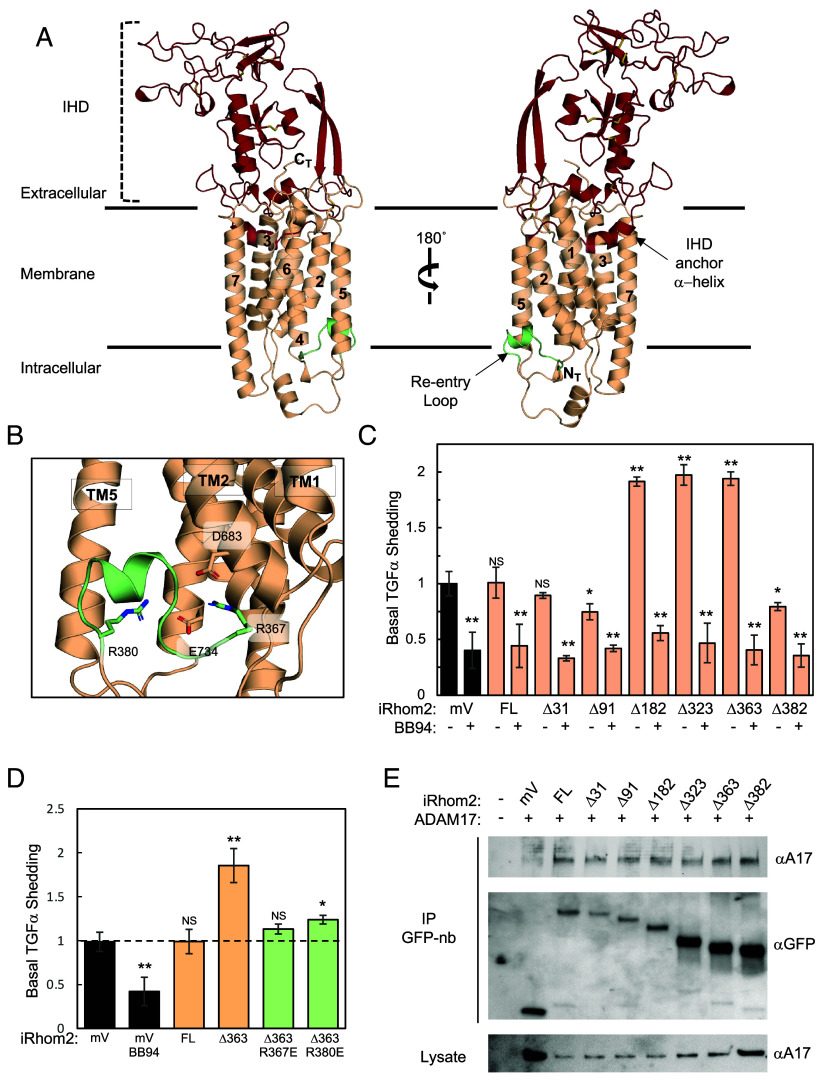
Analysis of the iRhom2 cytoplasmic and reentry loop in ADAM17 activity. (*A*) Cartoon representation of iRhom2 within the cell membrane. The IHD is highlighted in crimson and indicated with a dashed bracket. The transmembrane (TM) α-helices are numbered sequentially (1 to 7), and the reentry loop, shown in green, is marked with an arrow positioned between TM2 and TM5. (*B*) Close-up view of the reentry loop with the residues R367, R380, D683, and E734 displayed as sticks and color-coded in RGB. (*C*) Basal shedding assay of TGFα mediated by ADAM17. U2OS cells were cotransfected with AP-TGFα and either mVenus (mV), full-length iRhom2 (FL), or iRhom2 constructs with cytoplasmic truncations. Normalized AP-TGFα shedding (N ≥ 3) was quantified with and without the panmetalloproteinase inhibitor BB94. All iRhom2 transfections were normalized to the mVenus control in the absence of BB94. (*D*) Basal shedding assay of TGFα mediated by ADAM17 with reentry loop mutations. U2OS cells were transfected with AP-TGFα and either mVenus (mV), full-length iRhom2 (FL), Δ363-iRhom2, Δ363-iRhom2 R367E, or Δ363-iRhom2 R380E. AP-TGFα shedding (N ≥ 3) was normalized to the mVenus control. Error bars represent mean ± SD of independent experiments. Statistical significance was determined using an unpaired, two-tailed *t* test between the mV sample and each iRhom2 sample. *P < 0.05; **P < 0.005; NS, not significant. (*E*) Immunoprecipitation (IP) analysis of mVenus–iRhom2 constructs with ADAM17. Expi293F cells were cotransfected with catalytically inactive ADAM17 E406A and either mVenus (mV), full-length iRhom2 (FL), or iRhom2 constructs with cytoplasmic truncations. Lysates immunoprecipitated using GFP nanobody (GFP-nb) resin were analyzed by western blot for ADAM17 (*Top* panel) and iRhom2-mVenus (*Middle* panel). Total ADAM17 levels in cell lysates were confirmed by western blot (*Bottom* panel).

iRhom2 has evolved from the rhomboid superfamily into a pseudoenzyme that lacks enzyme activity and features a large extracellular IHD. The IHD is a section of 243 amino acids nestled between TM1 and TM2, anchored into the cell membrane by an amphipathic α-helix preceding TM2 (Fig. 3*A*). This domain is stabilized by a network of 8 disulfide bonds, extending 45 Å from the cell surface, and capped by an unstructured region responsible for tethering the ADAM17 ECD (Figs. 1 and 3*A*). The iRhom2 TM1-6 α-helices fold into a rhomboid-like domain with TM7 located to the periphery of the rhomboid-like α-helical bundle (Fig. 3*A*). The prototypical rhomboid protease, GlpG (RCSB PDB: 2IC8), superimposes well (RMSD = 2.36 Å) onto the core iRhom2 TM1-4 and TM6 (SI Appendix, Fig. S4*A*). Interestingly, the iRhom2 TM5 is displaced from the core rhomboid fold by 12 Å to accommodate the insertion of a conserved iRhom2 cytoplasmic amphipathic α-helix (SI Appendix, Fig. S4 *B* and C), dubbed the “reentry” loop, into the base of the rhomboid-like fold (Fig. 3*B*).

The cytoplasmic domain of iRhom2 is crucial for regulating the stimulated shedding of ADAM17 substrates from the cell surface. To evaluate the role of the reentry loop in ADAM17 activity, we tested iRhom2 cytoplasmic deletion constructs in a well-established cell-based assay measuring ADAM17 processing of the chimeric alkaline phosphatase-transforming growth factor α (AP-TGFα) reporter (20, 60) (Fig. 3 *C* and *D*). In these assays, AP-TGFα shedding was assessed in the presence and absence of the metalloproteinase inhibitor BB94 to determine the contribution of ADAM17 activity to basal shedding. Deleting the cytoplasmic region of iRhom2, specifically up to amino acid residue M183 (Δ182), led to an increase in basal shedding of AP-TGFα compared to the overexpression of full-length iRhom2 (Fig. 3*C*), which is also seen with larger deletions, resulting in a shorter cytoplasmic domain (Δ323, Δ363). In contrast, deletion of the entire cytoplasmic region, including the reentry loop (Δ382), nearly abolished basal shedding of AP-TGFα (Fig. 3*C*), suggesting that the highly conserved reentry loop is crucial for the regulation of ADAM17 (Fig. 3*B* and SI Appendix, Fig. S4*C*). The reentry loop, spanning R367 and R380, is stabilized by these conserved residues through salt bridge interactions with E734 in TM4 and D683 in TM2, indicating its likely functional importance. To test this idea, we generated R367E and R380E mutations, which reverse the charge potential and attenuated the increased basal shedding activity observed with the Δ363-iRhom2 construct (Fig. 3*D*). Importantly, mutations within the reentry loop or its complete removal do not affect ADAM17 binding in co-IP experiments (Fig. 3*E*), underscoring the reentry loop of iRhom2 as a key cytoplasmic regulatory element that controls extracellular ADAM17-dependent substrate processing.

### Structure–Function Studies of the ADAM17–iRhom2 Contact Interface.

**Fig. 4.**
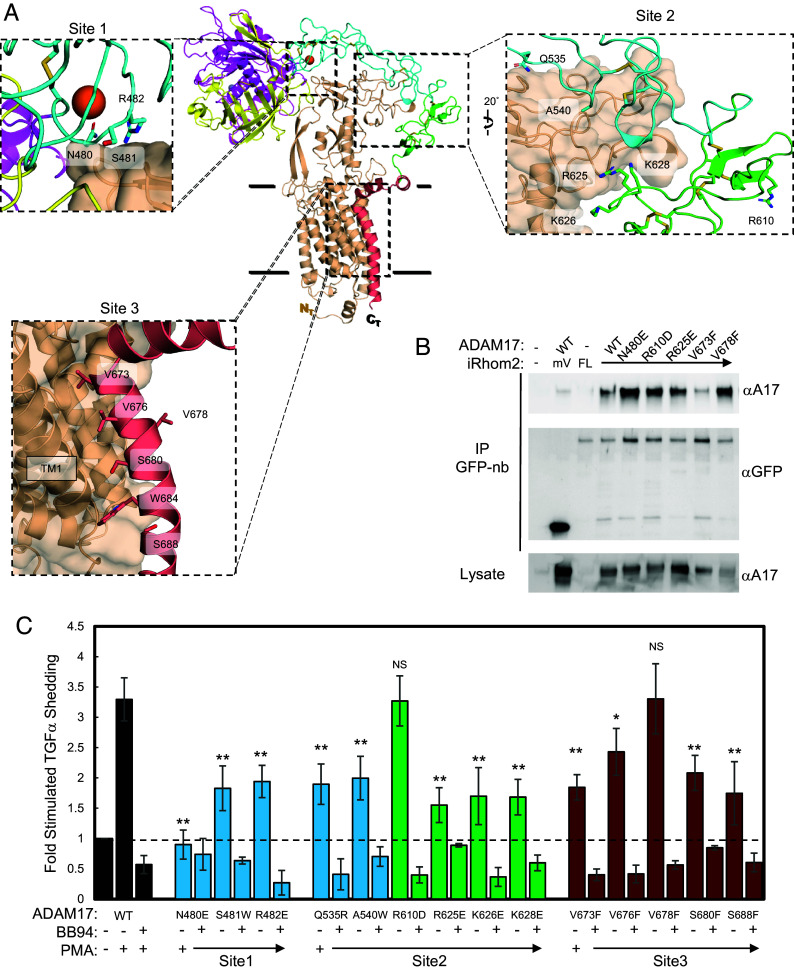
Analysis of the contribution of the iRhom2–ADAM17 contact interface to ADAM17 activity. (*A*) Cartoon representation of the ADAM17–iRhom2 complex showing three contact interfaces, designated as Sites 1–3. Zoomed views (*Inset* boxes) highlight the amino acid residues at each interface, with side chains displayed as sticks. These residues were assayed for their effects on ADAM17 activity. (*B*) IP analysis of mVenus–iRhom2 interaction with ADAM17 mutations. Expi293F cells were cotransfected with either mVenus, full-length mVenus–iRhom2, or ADAM17 constructs (WT or Site 1–3 mutants). Lysates were immunoprecipitated using GFP nanobody (GFP-nb) resin and analyzed by western blotting for ADAM17 (*Top* panel) and mVenus–iRhom2 (*Middle* panel). Total ADAM17 levels in lysates were confirmed by western blot (*Bottom* panel). (*C*) Stimulated shedding assay of TGFα mediated by ADAM17. *ADAM17^−/−^* cells were cotransfected with AP-TGFα and either WT ADAM17 or ADAM17 mutants. PMA-stimulated and BB94-treated samples were normalized to the unstimulated sample for each ADAM17 variant. Data are color-coded as follows: WT ADAM17 (black), D domain mutations (cyan), C domain mutations (green), and TM domain mutations (crimson). Error bars represent mean ± SD from *N* ≥ 3 independent experiments. Statistical significance, comparing PMA-stimulated samples, was determined using an unpaired, two-tailed *t* test to compare individual mutant samples to the WT control under stimulated conditions. *P < 0.05; **P < 0.005; NS, not significant.

The zymogen ADAM17–iRhom2 complex is stabilized by noncovalent interactions that can be divided into two distinct regions: one involving the ECDs and the other between the TM α-helices within the cell membrane (Fig. 4*A*). Surface charge potential analysis of iRhom2 revealed that the ECD is dominated by ionic interactions, notably through a negatively charged region within the IHD, which is proximal to a cluster of oppositely charged residues (R625, K626, and K628) in the ADAM17 C domain (Fig. 4*A* Site 2 and SI Appendix, Fig. S5 *A* and B). These amino acids in ADAM17 are functionally significant, as they were previously implicated in controlling ADAM17 enzyme activity required for normal embryonic development in mice (61).

To evaluate the significance of ADAM17 ECD positioning on the iRhom2 HD, we introduced point mutations at the contact points (Fig. 4*A* Site 1 and Site 2) in the ADAM17 ECD and assessed their effects on ADAM17 maturation and the stimulated shedding of AP-TGFα using the ADAM17 activator, phorbol 12-myristate 13-acetate (PMA) (20, 62). Among the tested mutations, R625E, R626E, and K628E at Site 2 caused a significant decrease in AP-TGFα processing upon stimulation (Fig. 4*C*). In contrast, the R610E mutation that is outside the iRhom2 HD binding interface, did not affect AP-TGFα processing and functioned similarly to wild-type ADAM17. Despite the D and C domains of ADAM17 having few secondary structural features, they are stabilized and rigidified by a network of 13 disulfide bonds. Notably, the N480E mutation in site 1, located approximately 40 Å from R625 on the opposite side of the iRhom2 HD interface, completely abolished AP-TGFα-stimulated shedding (Fig. 4*C*). All mutant ADAM17 proteins still underwent maturation, a process dependent on iRhom binding (SI Appendix, Fig. S5*D*), and bound to iRhom2 comparable to the WT control (Fig. 4*B* and SI Appendix, Fig. S6). These findings support a model in which the ancillary domains of ADAM17 serve as a rigid scaffold, stabilized by the iRhom HD, to regulate ADAM17 function, perhaps through allosteric control of the M domain activity, alignment with substrate cleavage site, or both.

Conservation analysis of ADAM17 revealed strong preservation in the catalytic cleft and a continuous interface extending down its TM α-helix, suggesting an evolutionarily conserved functional significance for these residues (Fig. 4 Site 3 and SI Appendix, Fig. S5 *A* and C) (63). While iRhom2 exhibits low overall surface conservation, strong conservation is observed in TM1 and portions of the IHD hinge that interact with the ADAM17 TM α-helix (SI Appendix, Fig. S5*C*). To explore the role of this conserved TM interface, point mutations were introduced into the ADAM17 TM α-helix, and the stimulated shedding of AP-TGFα was evaluated (Fig. 4*C*). Individual mutations of the ADAM17 TM attenuated AP-TGFα shedding from the cell surface with V673F, S680F, and S688F having the most significant effect (Fig. 4*C*). In contrast, a mutation (V678F) located on the opposite face of the ADAM17 TM α-helix relative to iRhom2 interaction, showed no defects in stimulated shedding of AP-TGFα. In addition, ADAM17 carrying the TM V673F mutation exhibited a marked deficiency in co-IP with iRhom2 as compared to WT ADAM17 and other ADAM17 ECD mutations (Fig. 4*B* and SI Appendix, Fig. S6). These findings emphasize the critical role of the transmembrane interface in binding ADAM17 and facilitating its functional regulation as part of the complex.

### Prodomain Removal is a Prerequisite for ADAM17 Sheddase Activity.

**Fig. 5.**
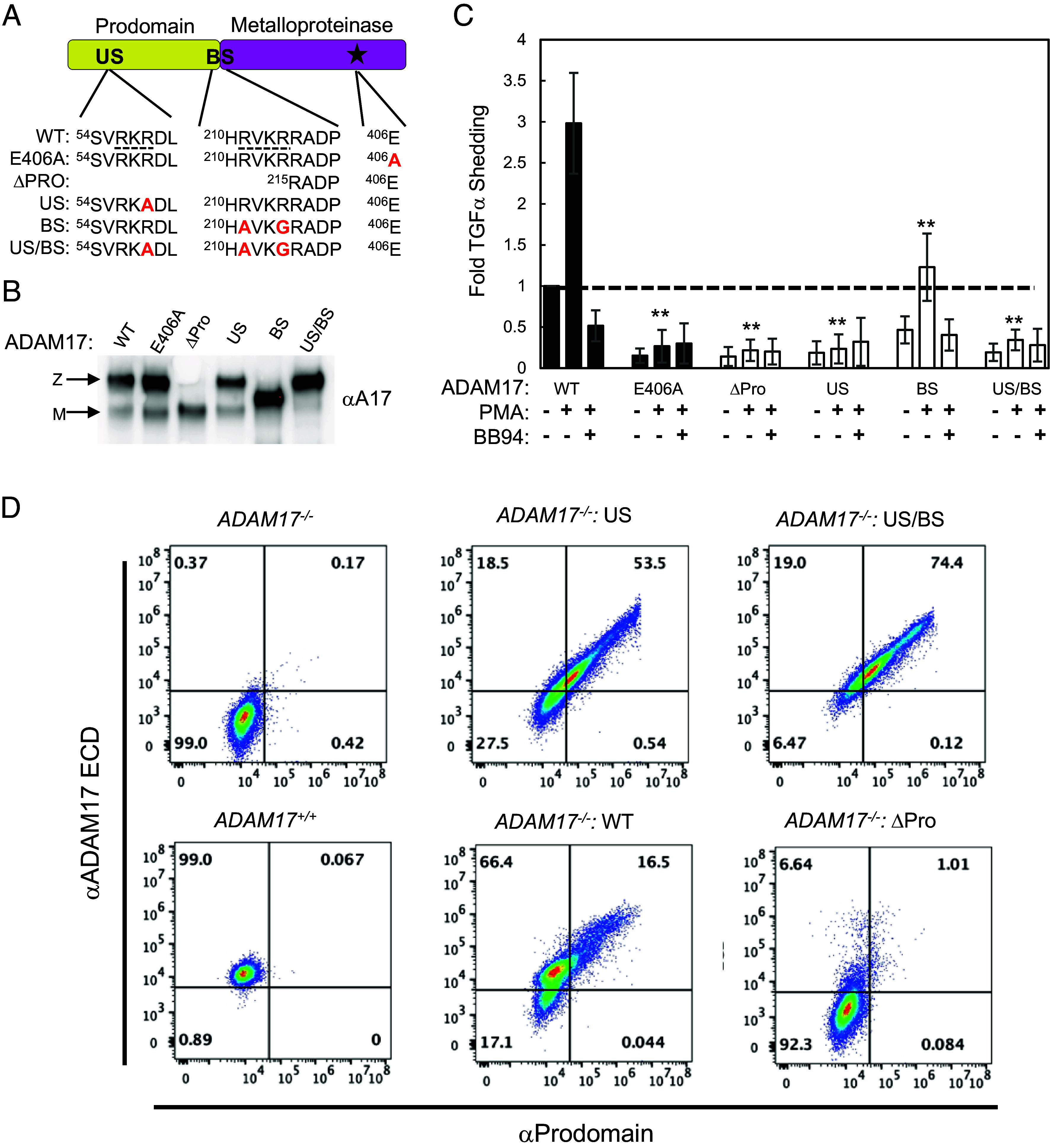
Analysis of prodomain processing in ADAM17 activity. (*A*) Domain organization of the ADAM17 Prodomain and metalloproteinase domain, highlighting the positions of the US, BS, and active site (depicted as a star), with the sequence alignments for the expression constructs. The consensus proprotein convertase processing sites are underlined with the point mutations to block maturation and enzyme activity indicated in red. (*B*) Western blot analysis of ConA-enriched lysates from cells transfected with ADAM17 variants: catalytically inactive E406A, ΔProdomain, US, BS, and US/BS mutations. To further distinguish between the zymogen (Z) and mature (M) forms of ADAM17, indicated with arrows, samples were treated with PNGase F. (*C*) Stimulated shedding assay of TGFα mediated by ADAM17. *ADAM17^−/−^* U2OS cells were cotransfected with AP-TGFα and either WT ADAM17 or ADAM17 prodomain mutants, as indicated. Shedding assays were conducted in the presence and absence of the panmetalloproteinase inhibitor BB94 to measure stimulated shedding activity. Data were normalized to the unstimulated WT ADAM17 control, in the absence of BB94. Error bars represent the mean ± SD from *N* = 4 independent experiments. Statistical significance of PMA-stimulated mutant samples compared to WT was assessed using an unpaired, two-tailed *t* test: *P < 0.05; **P < 0.005; NS, not significant. (*D*) Flow cytometry analysis of cell surface ADAM17 using selective antibodies targeting the prodomain and ectodomain. WT U2OS cells were compared to *ADAM17^−/−^* U2OS cells transfected with either WT ADAM17 or maturation-deficient ADAM17 mutants, including US/BS, US, and ΔProdomain. Quadrant gates were established based on the fluorescence signals from *ADAM17^−/−^* U2OS cells, which served as the negative control.

The conversion of zymogen ADAM17 to its functionally mature state proceeds through the proteolytic processing by proprotein convertases at two locations within the prodomain: the first at residue R58, designated the “upstream” processing site (US), and the second at the boundary site (BS) between the pro- and M domains (24, 25) (Figs. 1*A*, 2*A*, and 5*A* and SI Appendix, Fig. S1*A*). In our cryo-EM structures, the BS and preceding region leading to the inhibitory cysteine-switch are missing in the density map, presumably due to high flexibility imposed by the binding of the MEDI3622. However, the US is positioned proximal to and is stabilized by the iRhom2 HD (Fig. 1*A*). The stabilization of the US loop is underscored by its absence in the isolated cryo-EM structure of the MEDI3622 F_ab_-ProM domain (SI Appendix, Fig. S3*B*).

To investigate the role of prodomain removal in ADAM17 activation, mutations were introduced at the US site (R58A) and the BS (R211A/R214G), in order to ablate furin processing and prevent ADAM17 maturation (Fig. 5 *A* and *B*). The impact of these mutations on ADAM17 proteolysis and maturation were assessed using the AP-TGFα shedding assay (Fig. 5*C*). We found that mutation of the US site, either alone or combined with the boundary site, had the same effect as the inactivating catalytic site mutant (E406A) on the basal and stimulated shedding of TGFα from the cell surface (Fig. 5*C*). Mutating the BS site only reduced, but did not abolish TGFα shedding. Finally, expression of a mature form of ADAM17 lacking the entire prodomain (ΔPro) failed to elicit TGFα shedding (Fig. 5*C*). These findings indicate that the presence of the prodomain during biosynthesis and cleavage at the US site is required for the stimulated shedding by ADAM17 in cells.

To further investigate the mechanistic defects caused by the US mutation, we used flow cytometry to detect the levels of the ECD and prodomains of endogenous ADAM17 and overexpressed WT and US mutant ADAM17 forms at the cell surface. Surface staining of the US and US/BS ADAM17 mutants revealed strong correlation between cells stained with both an ADAM17 ECD antibody and an ADAM17 prodomain antibody (Fig. 5*D* and SI Appendix, Fig. S7). Notably, expressing wild-type ADAM17 in ADAM17-null cells revealed that only cells with the highest ADAM17 ECD staining were double-stained with the prodomain antibody. Furthermore, cells expressing endogenous ADAM17 exhibited lower surface levels of ADAM17 compared to WT-transfected cells, with no prodomain detected on the cell surface. Moreover, the ΔPro ADAM17 mutant showed little surface staining of ADAM17 ECD, suggesting that it suffered from significant trafficking defects to the cell surface (Fig. 5*D* and SI Appendix, Fig. S7). Taken together, this analysis supports a molecular model in which the prodomain is required for proper folding and exit of the ADAM17–iRhom2 complex from the ER. Pro-ADAM17 undergoes processing by proprotein convertases, such as furin, in the trans-Golgi network, resulting in dissociation of the prodomain from mature endogenously expressed ADAM17 prior to reaching the cell surface.

## Discussion

Here, we present the cryo-EM structure of the zymogen ADAM17 bound to iRhom2. The structure reveals critical interactions between the transmembrane α-helices and ECDs of both proteins. Moreover, it highlights a central role for the cytoplasmic reentry loop in iRhom2 as a regulator of ADAM17 function, which was further corroborated by cell-based studies. Overall, our findings offer detailed insights into the regulatory mechanisms controlling ADAM17 maturation and activity.

In our investigation, we found that the iRhom2 cytoplasmic region was a key feature in regulating enzyme function and in generating the zymogen ADAM17–iRhom2 complex that was amenable for structure determination. Phosphorylation of the iRhom2 cytoplasmic domain modifies its interactions with 14-3-3 adaptor proteins, which have emerged as key regulators of ADAM17 activity (40, 41, 43, 44). Additionally, the FERM domain-containing protein 8 (FRMD8, also known as iTAP), which interacts with the cytoplasmic domain of iRhom2, has been identified as a key regulator of ADAM17 function (40, 41, 43, 44). Consistent with this observation, FRMD8 knockout mice exhibit minimal mature ADAM17, aligning with the notion that FRMD8 is crucial for the proper assembly of the ADAM17–iRhom2 complex, thereby enabling ER exit (64). Moreover, gain-of-function point mutations (I186T or P189L) in a conserved cytoplasmic sequence in iRhom2 were identified as the cause for tylosis with esophageal cancer (TOC) (65). Interestingly, CUB (curly coat and bare skin) mice, which express a mutant form of iRhom2 lacking its N-terminal cytoplasmic sequences, including the FRMD8/iTAP-binding region and the conserved TOC region, have no detectable mature ADAM17–iRhom2 (45, 66–68). In macrophages lacking iRhom2, or in other tissues devoid of both iRhom1 and iRhom2, there is no detectable mature ADAM17, only a stable proform of ADAM17 is present, likely residing in the ER (32–35). Conversely, in the absence of ADAM17, iRhom2 stability is significantly compromised, further supporting the notion that heterodimeric complex formation is critical for the stability of iRhom2 (48). Interestingly, Lu et al. reported that coexpression of FRMD8 was vital for generating recombinant protein suitable for their structural studies of ADAM17–iRhom2 (49). In our study, we found that removing part of the iRhom2 cytoplasmic domain, including the FRMD8-binding site (41, 44, 64) was a crucial step in producing a stable ADAM17–iRhom2 complex that was amenable to structural characterization. Taken together, these findings suggest that binding of FRMD8 or deletion of its binding site on iRhom2 potentially facilitates the proper assembly and folding of the ADAM17–iRhom2 complex in the ER, which appears to be a prerequisite for ER exit and for generating a protein complex suitable for structural determination.

We and others have shown that proper ADAM17 trafficking and function require initial translation as a zymogen with an intact prodomain, followed by maturation involving the cleavage and removal of the prodomain in the trans-Golgi network (24, 54, 55). The importance of the prodomain is underpinned by its central position within the zymogen ADAM17–iRhom2 cryo-EM structure, which shows that the prodomain bridges the ADAM17 M domain to the iRhom2 HD. Indeed, interactions between zymogen ADAM17 and iRhom2 have been observed to be more stable than those of the mature ADAM17–iRhom2 complex (33). As this manuscript was in preparation, a cryo-EM structure of the zymogen ADAM17–iRhom2 complex was published that readily superimposes (RMSD = 0.813 Å) onto the structure presented here (49). Lu et al. proposed that the release of the processed prodomain represents the mechanism underlying ADAM17 activation by stimuli, such as the phorbol ester PMA. However, this interpretation is based on the properties of a mutant ADAM17 in which the US site cannot be processed, similar to the construct described above and to a previously reported construct that does not exhibit sheddase activity toward TGFα (25). Here, we confirm that mutating the US site severely attenuates ADAM17 sheddase activity. Furthermore, we show that the fully processed prodomain does not remain associated with mature endogenous ADAM17, suggesting that it is rapidly removed following processing by a proprotein convertase in the trans-Golgi network. Thus, since the prodomain cannot be detected on the surface of unstimulated wild-type cells, it is unlikely to regulate ADAM17’s rapid activation in response to stimuli. Instead, we propose that ADAM17 activation depends on allosteric conformational changes in the ADAM17–iRhom2 complex that are controlled by the iRhom2 reentry loop.

Comparative analysis of the ADAM17–iRhom2 and ADAM10–tspan15 complexes suggests that these multitransmembrane binding partners serve to tether the ancillary ADAM domains to the cell surface such that they assume a similar conformation, potentially restricting the conformational states of the ADAM extracellular region. Both ADAM17 and ADAM10 use cysteine-rich domain interactions to engage their binding partners’ ECD, but functional differences emerge upon disruption of these interactions. For ADAM10, ablation of this binding significantly reduced Tspan15 co-IP and impaired ADAM10 maturation. In contrast, while this region plays an important functional role in regulating ADAM17, mutations here did not affect ADAM17 maturation or its ability to bind iRhom2. This difference is likely due to the strong evolutionary interaction between ADAM17 and iRhom2 at the TM domains, which provides additional stability. Unlike ADAM17, ADAM10 likely does not exhibit strong TM domain interactions with Tspan15, and overexpression of this region in cells did not result in dominant-negative activity toward receptor shedding, as observed with the overexpression of the ancillary domains (59). While both ADAM10 and -17 complexes share structural similarities, particularly in how the multipass transmembrane binding partner ECD bridges the ADAM ancillary domains to the M domain, the ADAM17–iRhom2 complex is distinguished by its more extensive contact interface in the ECD regions and critical interactions within the TM domains of ADAM17 and iRhom2.

While the structure of mature ADAM17 bound to iRhom2 remains to be solved, our structure and mutational analysis support a model in which proprotein convertase–dependent removal of the prodomain allows the mature ADAM17 to interact with the iRhom2 HD in a way that ensures the correct processing of membrane tethered substrates (52). In agreement, the Dusterhoft group showed that a W567S mutation, buried in the iRhom2 HD, likely compromises the IHD’s structural integrity and its binding to the ADAM17 ECD, thereby attenuating ADAM17 function in cells without disrupting complex formation with iRhom2 (69). Moreover, we show that key ADAM17 residues (R625, K626, and K628) in the C domain interact with an oppositely charged surface on the iRhom2 HD, positioning the ADAM17 ECD relative to the cell surface. It is tempting to speculate that binding proteins for both ADAM10 and ADAM17 serve to position the rigid C + D domains, thus aiding in the precise positioning of the M domain. This restriction could function akin to a molecular ruler, as described for ADAM10 (52), or as a conformational scaffold for the catalytic domain, as suggested for the iRhom2–ADAM17 complex (70), contributing to their substrate selectivity. This suggests that therapeutic targeting of the ADAM17–iRhom2 ECD interface, particularly the iRhom2 HD, could be a viable strategy for inhibiting ADAM17 activity. This approach may create more selective inhibitors than antibodies targeting the ADAM17 protease directly, as anti-iRhom2 antibodies would not interfere with ADAM17–iRhom1 complexes. This selectivity could be especially relevant in chronic inflammatory diseases such as rheumatoid arthritis and systemic lupus erythematosus glomerulonephritis, where dysregulated ADAM17–iRhom2 activity plays a critical role (5, 6).

Stimulated ADAM17 activity is dependent on complex formation with iRhom2, which requires the TM helices and specific regions of the iRhom2 cytoplasmic domain (28, 40, 41, 43, 44, 46). The TM regions of ADAM17 and iRhom2 form a conserved interaction site that regulates ADAM17’s initial assembly and activity. Swapping ADAM17’s TM with that of an unrelated type-1 TM α-helix from BTC or CD62L abolishes stimulated activity, whereas restoring ADAM17-specific TM residues reinstates activity (47). Prior studies have shown that the cytoplasmic domain of ADAM17 is dispensable for rapid activation by various stimuli, positioning iRhom proteins as key regulators of ADAM17 activity (26, 28). Our finding that a conserved cytoplasmic reentry loop near TM1 affects ADAM17 activity provides a possible mechanistic explanation. Constructs of iRhom2 with nearly complete cytoplasmic deletion but retention of the reentry loop (Δ363) activate ADAM17-dependent shedding without stimulation. This activation is lost with mutations in the reentry loop (R367E and R380E) or deletion of the reentry loop (Δ382). Intriguingly, the reentry loop is positioned within the cytoplasmic facing core rhomboid fold of iRhom2 between TM2 and TM5, which in rhomboid protease proteins typically create a substrate-binding pocket guiding substrates to the active site. In contrast, iRhom2 binds ADAM17 primarily along the outward-facing side of TM1, leaving the TM2–TM5 interface unobstructed. The functional relevance of TM2 is further underscored by missense mutations in mouse iRhom2 TM2 (V645E and L655Q, aligning with human iRhom2 V674 and L684) that impair the proteolytic processing of TNFR2 and TNFα (71, 72). Furthermore, deleting nine residues in the iRhom2 HD near TM2 abolishes iRhom2-dependent ADAM17 activity in iRhom1/2^−/−^ knockout cells (47). Finally, a mutation in the TM2 of iRhom1 (G665W, aligning with iRhom2 G666) has been linked to cardiomyopathy in a patient (73). Collectively, these results indicate that the reentry loop is crucial for regulating ADAM17 activity by iRhom2, likely by affecting the relative positions of TM2 and TM5. We speculate that the TM2 may act as a fulcrum, transmitting positional changes across the membrane to affect the orientation of the extracellular iRhom2 HD, which resides between TM1 and TM2. Furthermore, changes in the iRhom2 HD could, in turn, modulate ADAM17 function, explaining why ADAM17’s cytoplasmic domain is dispensable for activation while its TM domain is essential. Further studies are in progress to test these possible hypotheses.

### Limitations of the Study.

Despite these advances, certain limitations remain. Our structure lacks the cytoplasmic regulatory elements of iRhom2, including the FRMD8 binding sites, that are involved in controlling stimulated shedding. Future studies will be required to explore the possibility of distinct active and inactive conformations of the ADAM17–iRhom2 complex. This is highlighted by the conflicting results between our study and that of Lu et al., which examined mutations at comparable locations within the ADAM17–iRhom2 extracellular interface. Key differences may arise from whether mutations were introduced in iRhom2 (as in the study of Lu et al.) or in ADAM17 (as done here), as well as from the assays used to evaluate function. While Lu et al. assessed basal shedding, our study focused on stimulated shedding of an ADAM17-selective substrate. Furthermore, the molecular and atomic details underlying ADAM17 substrate processing and protein substrate binding and selectivity are still not fully understood. Moreover, the details of how exactly stimulation from inside the cell results in changes to ADAM17 activity in the extracellular space remain to be established. Finally, it will be important to determine the conformation of ADAM17 bound to iRhom1 by Cryo-EM, which would address whether these proteins might direct ADAM17 into different conformational states. Future research should aim to resolve these gaps, focusing on the regulatory mechanisms of iRhom proteins and the active states of the ADAM17–iRhom2 complex. Understanding these aspects could significantly enhance our ability to develop targeted therapies for diseases associated with dysregulated ADAM17 activity.

## Methods and Materials

Expression constructs encoding ADAM17, iRhom2, and the MEDI3622 F_ab_ were sequence-verified and expressed in mammalian systems. The ADAM17 ProM domain and the ADAM17–iRhom2 complex used for structure determination were produced in Expi293F and BacMam-infected Expi293F cells, respectively, and purified by affinity and size-exclusion chromatography under conditions optimized for cryo-EM. Cryo-EM grids were imaged at two facilities: The ADAM17–iRhom2 complex was imaged on a 300 kV Titan Krios microscope equipped with a K3 BioQuantum detector at the Vanderbilt University Center for Structural Biology, while the ADAM17 ProM Domain–MEDI3622 F_ab_ complex was imaged on a 200 kV Glacios microscope with a Falcon 4D detector at the University of Cincinnati Center for Advance Structural Biology. Image processing and single-particle analysis were performed using CryoSPARC (74) on the GPU cluster at the University of Cincinnati’s Advanced Research Computing Center. A detailed description of all experimental procedures is provided in SI Appendix, including protocols for expression construct design, protein production and purification, cryo-EM sample preparation, and image processing.

Cell-based AP-TGFα ectodomain shedding assays were performed in either wild-type and *ADAM17^−/−^* U2OS cells cotransfected with ADAM17, iRhom2, or control constructs. Shedding of TGFα was measured by alkaline phosphatase activity in the media and cell lysates following treatment with PMA, BB94, or both. Co-IP assays were carried out in Expi293F cells coexpressing ADAM17 and mVenus-tagged iRhom2, followed by IP with GFP nanobody resin and immunoblotting for ADAM17 and GFP. Flow cytometry was used to assess surface expression of ADAM17 and its prodomain (24), using fluorescence-based single-cell gating. Additional methodological details for the functional assays, including buffer compositions, transfection conditions, and statistical analysis procedures, can be found in SI Appendix.

## Supplementary Material

Appendix 01 (PDF)

## Data Availability

Structural data, including the maps and coordinate files, for the MEDI3622 F_ab_–ADAM17–iRhom2 and MEDI3622 F_ab_-ADAM17 Pro-M domain complexes are publicly available at the RCSB Protein Data Bank under PDB IDs 9O58 and 9O54 (75, 76), respectively (https://www.rcsb.org/) (77). All other data are included in the manuscript and/or SI Appendix.
