# Supplementary material for: Structural insights into the activation and inhibition of the ADAM17–iRhom2 complex
Source: Proc Natl Acad Sci U S A. 2025 Jun 13;122(24):e2500732122. doi: 10.1073/pnas.2500732122 (PMC12184519; doi:10.1073/pnas.2500732122)
Supplement: Supplementary file 1 — Appendix 01 (PDF) [file pnas.2500732122.sapp.pdf]

SUPPORTING INFORMATION

Structural Insights into the Activation and Inhibition of the ADAM17-iRhom2 Complex

Joseph J. Maciag[1]*, Conner Slone[1]*, Hala F. Alnajjar[1], Maria F. Rich[1], Bryce Guion[1], Igal Ifergan[1], Carl P. Blobel[2,3] and Tom C.M. Seegar[1,#]

[1]Department of Molecular and Cellular Biosciences, University of Cincinnati College of Medicine, Cincinnati, OH 45267, USA.

[2]Department of Medicine and of Biochemistry, Cell and Molecular Biology, Weill Cornell Medicine, New York, NY 10021, USA.

[3]Arthritis and Tissue Degeneration Program, Hospital for Special Surgery, New York, NY 10021, USA.

*Equal Contribution

[#]Correspondence to: tom.seegar@uc.edu

This File Includes:

Supplementary Methods Text

Supplementary Figures 1-7

Supporting Information References

SUPPLEMENTARY METHODS

Cell Lines

All cell lines used in this study (U2OS, Expi293, and SF9) have been described previously and were maintained in their respective media [1, 2]. The generation of the *ADAM17*$^{-/-}$ U2OS cell line was performed by the Cincinnati Children's Hospital Medical Center Transgenic Animal and Genome Editing Core Facility, which introduced a nonsense mutation into exon 2 of the *ADAM17* gene locus. Successful genome editing by CRISPR-Cas9 repair was validated through sequencing, and western blot analysis confirmed a complete loss of ADAM17 protein expression.

Expression Constructs

The ADAM17 expression vectors were subcloned from pRK5M-ADAM17myc (Addgene: Plasmid #31714) into the pEG BacMam expression vector (Addgene: Plasmid #160451) [3, 4]. The extracellular ADAM17 pro- and metalloproteinase domains (residues 1– 477) were cloned into the pLib vector (Addgene: Plasmid #80610), which includes a 6x HisTag at the carboxyl terminus [5]. All iRhom2 expression constructs were PCR-amplified from a cDNA (Origene) and sequentially subcloned into the p-mVenusC1 vector (Addgene: Plasmid #27794), followed by cloning into pEG BacMam as mVenus-iRhom2 chimeric proteins [6]. The MEDI3622 antibody F_{ab} fragment sequence was obtained from World Intellectual Property Organization Patent #WO 2016/089888 A1. Geneblock fragments

encoding the light and heavy chains were cloned into the expression vectors pD2610-v5 (ATUM) and pFUSE-hIgG1-Fc1 (Invitrogen), respectively, with the latter containing a 3C protease site upstream of the Fc region. The AP-TGFα reporter construct was generated by cloning a Geneblock encoding TGFα (residues 40–161) into the pTAG5-AP vector (GenHunter: Cat#QV5). All point mutations in ADAM17 and iRhom2 were introduced using overlapping PCR primers. The ∆Pro ADAM17 (amino acid 215-824) was inserted into the pRK5M-myc expression vector behind its native signal sequence using Infusion Cloning (Takara Bio Inc.). Each expression construct was validated by Sanger sequencing.

Recombinant Protein Production and Isolation

The heavy and light chains of the MEDI3622 antibody were co-transfected (1 μg/mL, 1:1 Light to Heavy Chain ratio) into Expi293F cells at a density of 2 × 10^6 cells/mL using a 1:1 DNA to 40 kDa polyethyleneimine HCl MAX ratio. After 24 hours, the cell culture was supplemented with 450 mg/mL of D-(+)-glucose and 5 mM valproic acid sodium salt. Transfected cultures were incubated at 37°C while shaking for a total of five days. Conditioned media was collected by centrifugation at 4000 rpm for 30 minutes and supplemented with 20 mM HEPES, pH 8.0, and 150 mM NaCl. The clarified media was applied to a Protein-A Sepharose column, extensively washed with 20 mM HEPES, pH 8.0, and 150 mM NaCl, and the bound protein was eluted in a 40 mM glycine, pH 3.0, and 150 mM NaCl buffer. The eluate was immediately neutralized with 1 M HEPES, pH 8.0. The MEDI3622 antibody was treated overnight at 4°C with 100 ng of 3C protease per

5 µg of antibody to release the F_{ab} fragment from the Fc region. The MEDI3622 F_{ab} fragment was purified using a Superdex 200 Increase (Cytiva) column equilibrated with 20 mM HEPES, pH 8.0, and 150 mM NaCl.

The A17 ProM Domain protein, containing the single point mutations N174Q, R58A, R211A and R214A, was expressed from baculovirus-infected *Spodoptera frugiperda*-derived ovarian cells (SF9) at a density of 3.0×10^6 cells/mL. Cultures were incubated at 27°C with shaking at 120 RPM for an additional four days. Conditioned media was collected by centrifugation and supplemented with 20 mM Tris buffer, pH 7.5, containing 150 mM NaCl, 5 mM $CaCl_2$, 1 mM $NiCl_2$, and 0.01 mM $ZnCl_2$. The media was clarified by centrifugation to remove residual debris and applied to a Ni-NTA column. The column was extensively washed with buffer containing 20 mM HEPES, pH 8.0, 100 mM NaCl, and 20 mM Imidazole and bound protein was eluted in the same buffer with 250 mM imidazole. Fractions were concentrated with centrifugal filters and buffer exchanged to remove excess salt and applied to an anion exchange and eluted over a 1 – 50% gradient of 20 mM HEPES, pH 8.0, 1000 mM NaCl. Fractions containing the ADAM17 ProM domains were concentrated and mixed with excess MEDI3622 F_{ab} and isolated to homogeneity with a Superdex 200 10/300 Increase (Cytiva) size exclusion chromatography column in 20 mM HEPES, pH 8.0, and 150 mM NaCl for use in cryo-EM sample preparation.

Full-length ADAM17 (E306A) and mVenus-iRhom2 (residues 364–824) proteins were co-expressed in baculovirus-infected Expi293F cells at a volumetric ratio of 1:3,

corresponding to 6% v/v of the total cell volume. After 24 hours, the infected Expi293F cultures were supplemented with 450 mg/mL D-(+)-glucose and 5 mM valproic acid sodium salt. The cells were harvested after 72 hours by centrifugation at 4,000 rpm for 30 minutes and resuspended in a homogenization buffer containing 20 mM HEPES at pH 8.0, 300 mM NaCl, 1% lauryl maltose neopentyl glycol (LMNG) (Anatrace), 0.1% cholesteryl hemisuccinate (CHS) (Anatrace), 1 mM $CaCl_2$, and 30% glycerol. The suspension was homogenized by stirring at 4°C for one hour. Cellular debris was removed by centrifugation at 14,000 rpm for 45 minutes, and the resulting supernatant was filtered through a 0.7 µm glass microfiber filter (Watman). The clarified supernatant was applied to a CNBr-GFP Nanobody affinity column and extensively washed with two column volumes of a buffer containing 20 mM HEPES at pH 8.0, 300 mM NaCl, 0.1% LMNG, 0.01% CHS, 0.1 mM $CaCl_2$, and 3% glycerol. To exchange detergents, the bound protein was sequentially washed with an exchange buffer containing 20 mM HEPES at pH 8.0, 150 mM NaCl, and 0.1% glycol-diosgenin (GDN) (Anatrace), followed by a second buffer containing 20 mM HEPES at pH 8.0, 150 mM NaCl, and 0.05% GDN. The protein was eluted using a buffer composed of 100 mM glycine at pH 3.0, 150 mM NaCl, and 0.05% GDN and was immediately neutralized with 10 mL of a neutralizing buffer containing 450 mM HEPES at pH 8.0, 150 mM NaCl, and 0.05% GDN. The eluted protein was concentrated using a 100 kDa centrifugal filter at 4,000 rpm until the total volume was reduced to 1 mL. The sample zymogen ADAM17-iRhom2 complex was mixed with MEDI3622 F_{ab} then applied to a Superose 6 Increase 10/300 GL (Cytiva) size-exclusion chromatography column, and fractions were analyzed by SDS-PAGE analysis and pooled

based on sample homogeneity. The pooled fractions were further concentrated and immediately used for cryo-EM sample preparation.

Cryo-EM Sample Preparation and Data Acquisition

The ADAM17 – iRhom2 and ProM domain in complex with the MEDI3622 F_{ab} were concentrated to 5.3 mg/mL and 0.25 mg/mL, respectably. These samples were individually applied to AltrAuFoil® R 1.2/1.3 grids that were glow discharged using a PELCO easiGlow™ Glow Discharge Cleaning System; 0.39 mBar, 20 mA, glow time/hold 30/10s. Grids were blotted with a blot force setting between 1-10, for times ranging 1-11s and plunge frozen in liquid ethane using a Vitrobot Mark IV System.

ADAM17 – iRhom2 grids were imaged at the Vanderbilt School of Medicine Center for Structural Biology on a FEI Titan Krios operated at 300 kV with a K3 BioQuantum direct electron detector camera in counting mode. Data sets were collected at a nominal magnification of 105,000x with a pixel size of 0.822 Å and defocus range between -0.4 to -2.2 µm. Two data sets were collected using a total dose of 62.2 $e^-/Å^2$ and 56.3 $e^-/Å^2$ were merged, totaling 24,828 movies, that were used for single particle analysis structure determination. The ADAM17 Pro-M Domain grids were imaged at the University of Cincinnati School of Medicine Center for Advanced Structural Biology on a Glacios operated at 200 kV with a Falcon 4D direct electron detector. A single data set, totaling 8,704 movies, was collected at a nominal magnification 165,000x with a pixel size of 0.69

Å, defocus range -0.4 to -2.0 μm and total dose of 40.0 $e^-/Å^2$ was used for structure determination.

Cryo-EM Single Particle Analysis and Model Building

All cryo-EM data were processed using CryoSPARC on the GPU cluster maintained by the University of Cincinnati Advanced Research Computing Center and all cryo-EM software, excluding CryoSPARC, was maintained in collaboration with SBGrid [7]. Movies were motion-corrected using patch motion correction, and contrast transfer function (CTF) parameters were estimated with patch CTF estimation. Movies were curated based on total full-frame motion distance (below 40 pixels), relative ice thickness (below 1.1), and CTF fit resolution (below 4.5 Å). For the ProM domain and ADAM17–iRhom2 structures, this process resulted in 5,673 and 18,680 movies, respectively, being selected for structure determination.

Initial particles were picked using a Laplacian of Gaussian autopicking approach on a subset of 300 curated movies to generate template 2D classes for particle picking across a larger dataset. For the ADAM17–iRhom2 complex, these 2D templates were used to pick an additional 2,084,443 particles from 5,652 curated movies. Iterative 2D classification reduced this set to 440,135 particles, which were used for iterative *ab initio* reconstruction, yielding an initial 6 Å density map for the ADAM17–iRhom2 complex. This particle stack served as a reference for training the Topaz particle-picking model, which subsequently identified 2,965,292 particles from the total curated movie set [8]. After further curation through 2D classification, 2,421,726 particles remained. These particles

underwent heterogeneous refinement using three input models, resulting in a 6.25 Å density map containing 250,122 particles. Iterative 3D classifications and local refinements improved the resolution to 3.53 Å. The overall workflow is depicted in Supplemental Figure 2. AlphaFold predictive models for the iRhom2 – ADAM17 zymogen complex and MEDI3622 F_{ab} were fit into the density using ChimeraX [9, 10]. Due to the slight structural reorganization of ADAM17 domains from the predictive AlphaFold model, the ADAM17 model was divided into three parts for fitting: the ProD domain, C + D domains and TM region. The model was built in Coot and refined in Phenix Real-Space Refine [11, 12]. Model to map FSC was produced using Phenix Comprehensive Validation (Table 1). The final map was post-processed with DeepEMhancer using the highRes algorithm for figure creation [13].

For the ProM domain, initial 2D classes were curated for use within the Topaz CryoSPARC workflow, resulting in 2,357,801 particles, which were reduced to 334,801 particles after 2D classification. This curated particle stack was used to generate three *ab initio* models, one of which represented the complete ProM-F_{ab} complex. These initial models underwent heterogeneous refinement against the 2,357,706-particle stack, followed by additional heterogeneous refinements to sort particles into the full complex map. The final density map was generated using a 3D-sorted particle set of 346,939 particles. Iterative *ab initio* modeling of two low-similarity classes (similarity score = 0.01), followed by NU-refinement and local refinement, resulted in a final density map with an overall resolution of 3.5 Å using 98,475 particles (Supplemental Figure 3). The coordinates for the Pro-M domain and MEDI3622 F_{ab} from the complex described above

were used to fit into the final density map using ChimeraX. This model was also built in Coot and refined in Phenix Real-Space Refine. Model to map FSC was produced using Phenix Comprehensive Validation (Table 1). The final map was post-processed with DeepEMhancer using the highRes algorithm and the overall workflow is depicted in Supplemental Figure 3.

ADAM17 Ectodomain shedding Assay

The methods for ADAM17 basal and stimulated ectodomain shedding assays were adapted from previously established protocols with slight modifications [14, 15]. In brief, wild-type (WT) or *ADAM17*$^{-/-}$ U2OS cells were seeded into 6-well tissue culture plates and co-transfected with either mVenus, ADAM17, or iRhom2 expression constructs along with the AP-TGFα reporter at a 1:2 ratio using PEI MAX. For stimulated shedding assays, 24 hours post-transfection, cells were washed with DMEM and serum-starved for 1 hr. Following this, the culture media was replaced with media supplemented with either 0.6% DMSO, 25 ng/μL PMA, or 5 μM BB94 + 25 ng/μL PMA. After 1 hr, conditioned media and cell lysates were collected, and the amount of AP activity in the supernatant and lysates was measured at an absorbance of 405 nm over a 90-minute time course following the addition of 1 mg/mL 4-nitrophenyl phosphate (New England Biolabs), using a BioTek SynergyH1 microplate reader. Ratios of AP activity in the supernatant to the combined cell lysate and supernatant were calculated. Each sample was normalized to its DMSO control to assess stimulation changes in response to the different pharmacological treatments. For basal shedding assays, 24 hr post-transfection, cells were washed, and

1 mL of DMEM supplemented with either 0.6% DMSO or 5 μM BB94 was added. After 24 hr, AP activity and ratios were calculated as described for the stimulated shedding assay. All sample ratios were normalized to the mVenus basal shedding.

Immunoprecipitation and Western Blotting

Expi293F cells were grown to a density of $1x10^6$ cells/mL and co-transfected with 1.5 μg of wild-type or mutant ADAM17 and 0.5 μg of mVenus-iRhom2 cDNA using Lipofectamine™ 2000 (ThermoFisher Scientific). After 24 hrs, cells were harvested by centrifugation at 14,000 RPM for 5 minutes and suspended in homogenization buffer containing 20 mM HEPES pH 8.0, 300 mM NaCl, 1% LMNG, 0.1% CHS, 1 mM $CaCl_2$, and 30% glycerol. The suspension was incubated for 1 hour. Lysates were clarified by centrifugation at 14,000 RPM for 5 min, and proteins were immunoprecipitated using CNBr-GFP Nanobody resin for 1 hour. The immunoprecipitation resin was extensively washed with 10x bead volume of homogenization buffer nine times to remove non-specifically captured proteins. Bound proteins were eluted by boiling the resin in 2x Laemmli Sample Buffer (BioRad #1610737), supplemented with 50 mM β-mercaptoethanol, for 5 minutes. The immunoprecipitated samples were analyzed by Western blot for the presence of ADAM17 and iRhom2-GFP using αTACE/ADAM17 (Cell Signaling, cat #6978) and αGFP (BioRad, cat #AHP975) antibodies, respectively.

Flow Cytometry

WT and *ADAM17*$^{-/-}$ U2OS cells were seeded in 6-well tissue culture dishes and transfected with 0.5 ug of the ADAM17 expression constructs, using PEI. After 48 hrs, cells were dislodged from the plate by trypsinization and isolated by centrifugation at 500 g for 5 minutes. Cells were stained for ADAM17 ECD and ADAM17 Prodomain with primary antibodies, αADAM17-alexafluor647 (R&D Systems) and αprodomain ADAM17 [16], respectively, at a 1:100 dilution for 90 minutes at room temperature in PBS supplemented with 0.5% m:v Bovine Serum Albumin. Cells were then washed with 10x volume of PBS and incubated for 60 minutes at room temperature with αRabbit-AlexaFluor488 (ThermoFisher) for detection of the prodomain antibody. Cells were subsequently washed with and resuspended in PBS for flow cytometry analysis. Single stain controls used for the ECD-ADAM17-alexafluor 647 and prodomain-αRabbit-AlexaFluor488 were WT U20S cells and *ADAM17*$^{-/-}$ U20S transfected with ADAM17US/BS mutations, respectively. Cell surface staining was assessed on a Cytek Aurora and analyzed by FlowJo software (v 10.10), measuring co-emission of 647 nm and 488 nm. For flow cytometry analysis, events were gated by FSC-SSC to remove debris and then restricted to single cells. Cells Quadrant gates for the purpose of quantification and depiction were generated with use of *ADAM17*$^{-/-}$ null cells.

Quantification and Statistical Analysis

Results of statistical analyses are found in the figure legends for Figures 3, 4, and 5. All calculations of significance were determined in Microsoft Excel software package using an unpaired t test (two-tailed). Data are reported as mean ± standard deviation.

Significance was determined by a p value of < 0.05, and annotated as $^{*}p < 0.05$ and $^{**}p < 0.005$. Not significant is annotated with NS.

SUPPLEMENTARY FIGURES

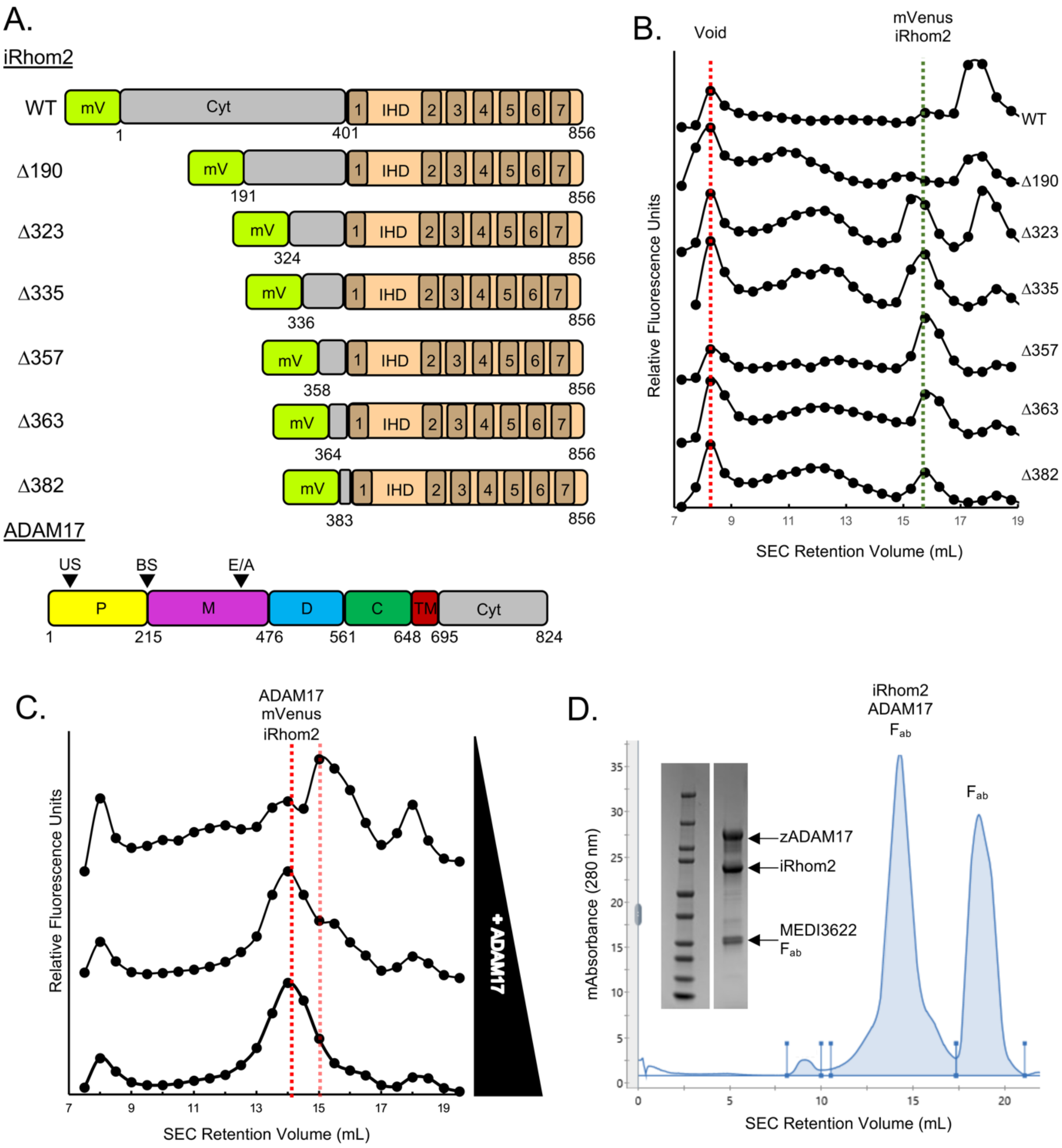

Supplementary Figure 1: Expression and Isolation of the Zymogen ADAM17-iRhom2 Complex. (A) Schematic representation with amino acid annotation of ADAM17 colored by domain and cytoplasmic deletion variants of the mVenus-iRhom2 proteins used in this study. The ADAM17 prodomain upstream (US) and boundary sites (BS), along with the catalytic E406A residue mutation (E/A) are indicated on the ADAM17

diagram. **(B)** Fluorescence-detection size exclusion chromatography (FSEC) of cytoplasmic truncated iRhom2 variants. The column void is indicated by the red line, and mVenus-iRhom2 is represented by the black line. **(C)** FSEC analysis of Δ363-iRhom2 co-expressed with increasing amounts of ADAM17. Peaks are annotated to indicate the formation of the ADAM17-iRhom2 complex with a leftward shift in the dashed red line. **(D)** Size exclusion chromatography (SEC) of the purified zymogen ADAM17-Δ363-iRhom2-MEDI3622 F_{ab} complex. Annotated peaks correspond to components of the complex. *(Inset)* Coomassie-stained SDS-PAGE shows the purity of the zymogen ADAM17, Δ363-iRhom2, and MEDI3622 F_{ab} complex used for cryo-EM sample preparation.

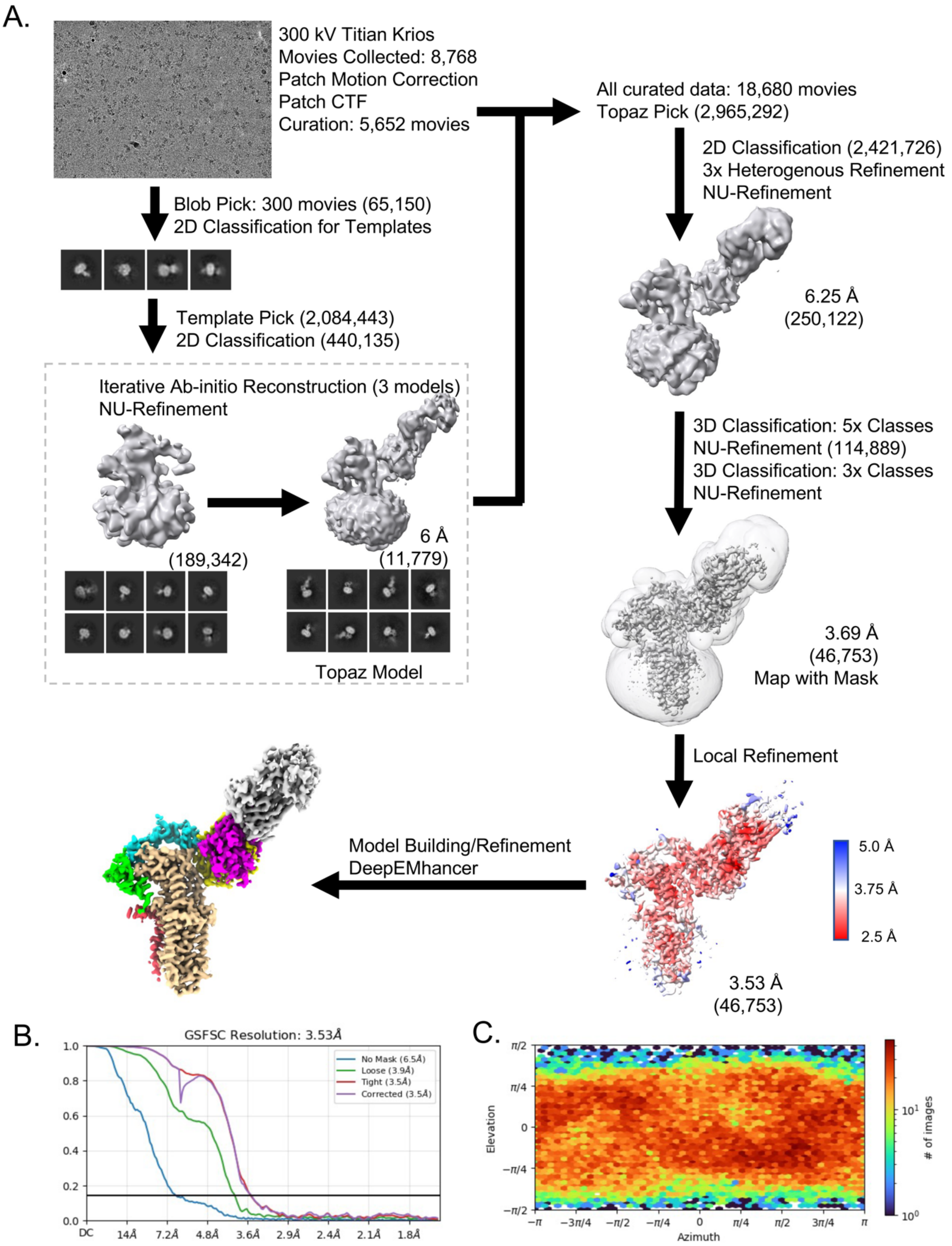

Supplementary Figure 2: Work Flow of the Structure Determination of the Zymogen ADAM17-iRhom2 Complex. (A) Workflow diagram illustrating the process used in

CryoSPARC to determine the structure of the zymogen ADAM17-Δ363-iRhom2-MEDI3622 F_{ab} complex. The particle numbers used are indicated in parentheses. **(B)** Fourier shell correlation (FSC) used to determine the overall resolution of the final density map. The black line marks the resolution corresponding to an FCS value of 0.143. **(C)** Angular distribution of the MEDI3622 F_{ab}-zymogen ADAM17-iRhom2 particles used in the final density map.

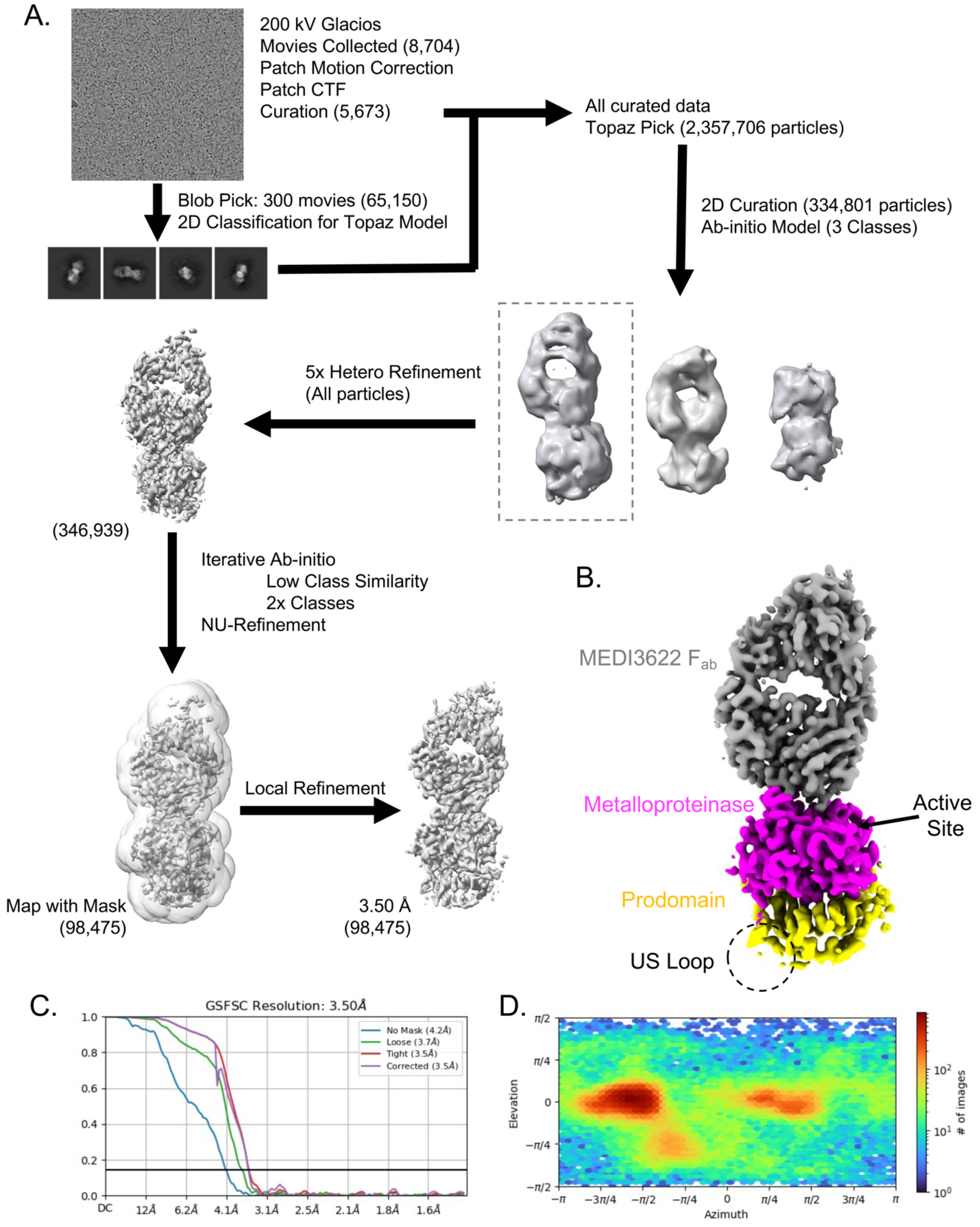

Supplementary Figure 3: Structure Determination of the ADAM17 Pro-M Domain

Workflow. (A) Workflow diagram illustrating the process used in CryoSPARC to determine the structure of the Prodomain and Metalloproteinase domain of ADAM17 in complex with the MEDI3622 F_{ab}. (B) DeepEMhancer sharpened density map of the ADAM17 ProM-MEDI3622 F_{ab} complex colored by domain as done in Figure 1. The missing density for the prodomain US pro-protein convertase site location is designed with a dashed circle. **(C)** Fourier shell correlation (FSC) used to determine the overall resolution of the final density map. The black line marks the resolution corresponding to an FCS value of 0.143. **(D)** Angular distribution of the MEDI3622 F_{ab}-ADAM17 Prodomain and M Domain particles used in the final density map.

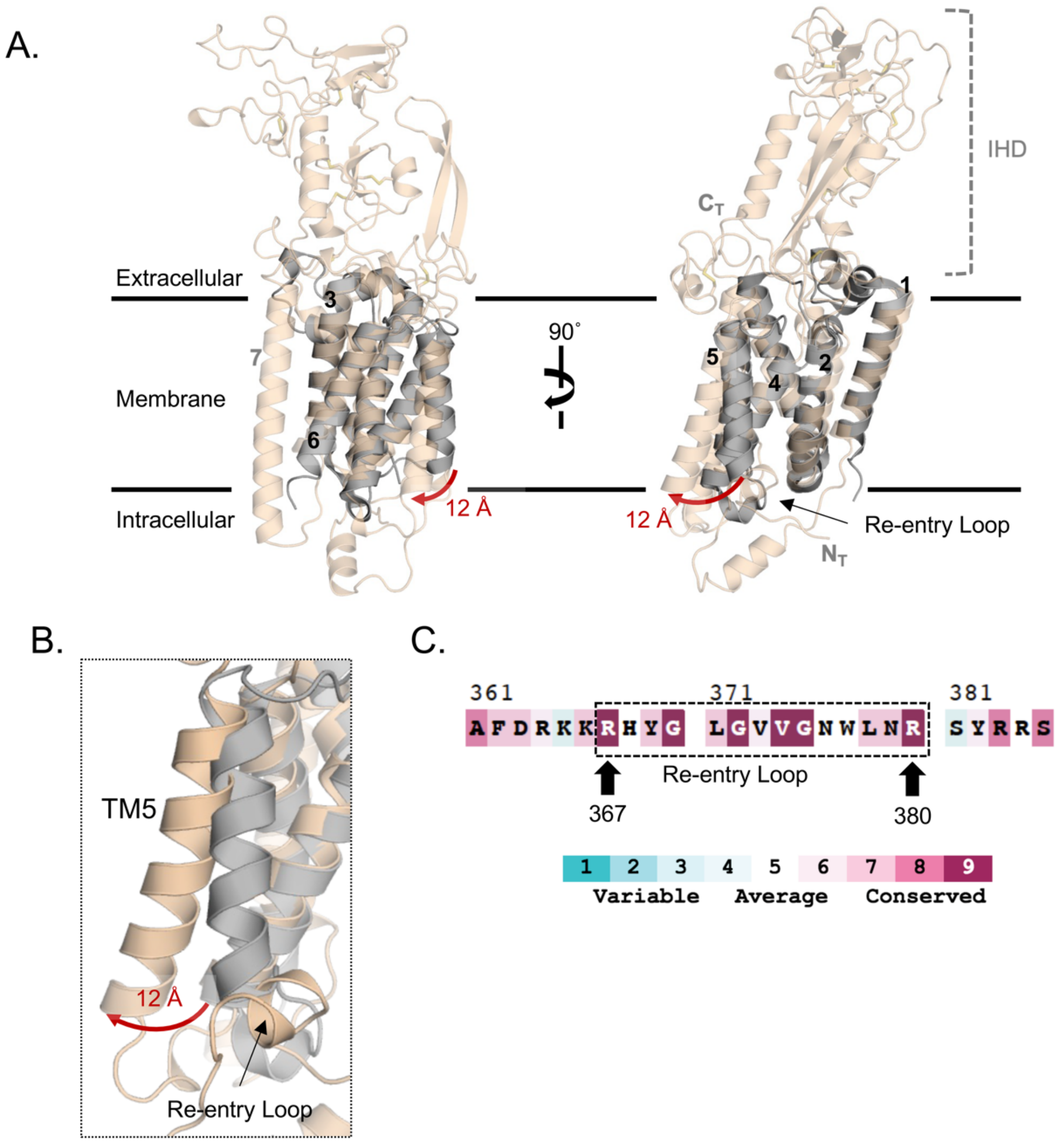

Supplementary Figure 4: Structural Elements of iRhom2 in ADAM17 Substrate Recognition. (A) Cartoon representation and structural superimposition of GlpG (dark gray; RCSB PDB: 2IC8) with iRhom2 (tan), shown embedded in the cell membrane. The re-entry loop of iRhom2 is annotated with an arrow, and the iRhom2 homology domain (IHD) is indicated with a dashed bracket. A red arrow highlights the displacement of iRhom2 TM5 relative to GlpG TM5 to accommodate the re-entry loop. **(B)** Magnified view

of the iRhom2 re-entry loop and TM5 of both iRhom2 and GlpC, showing a 12 Å movement in the iRhom2 TM5 that accommodate the re-entry loop relative to TM5 in GlpC. **(C)** Conservation analysis of iRhom2 residues 361–410 using the human iRhom2 sequence in the Consurf Server, displayed on a color-coded scale from variable (teal) to conserved (maroon) [17]. The re-entry loop is enclosed within a dashed box. Conserved Arg residues used in Figure 3D-E are annotated with arrows.

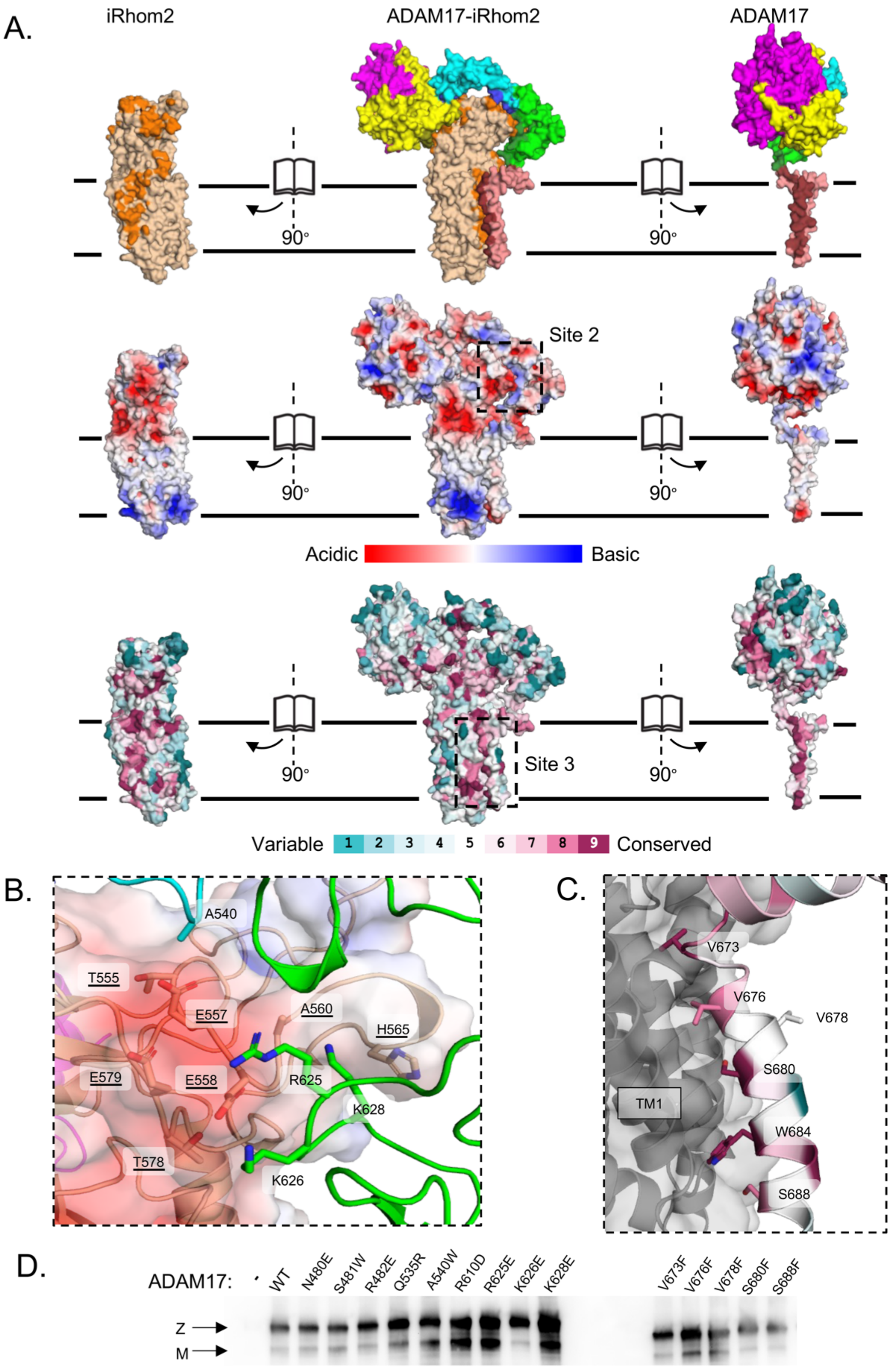

Supplementary Figure 5: Analysis of the Zymogen ADAM17-iRhom2 Contact

Interface. (A) Surface representation of the zymogen ADAM17-iRhom2 complex, with domains colored as in Figure 1. Open-book views illustrate the contact interface between zymogen ADAM17 and iRhom2. **Top:** The ADAM17-iRhom2 contact regions within 5 Å are highlighted with darker shades corresponding to their respective domains. Middle: Surface charge analysis, using the APBS tool in pymol, of the zymogen ADAM17-irhom2 complex, colored to indicate acidic residues in red and basic residues in blue. **Bottom:** Conservation analysis, using the Consurf Server, is mapped onto the surface of the complex, with scores represented on a gradient from teal (variable) to maroon (conserved). **(B)** Zoomed-in view of the Site 2 region from the electrostatic representation above, highlighting the ADAM17 residues A540, R625, K626 and K628. IHD amino acids within 5 Å of these ADAM17 residues are shown in stick format and numbered (underlined) according to their position in iRhom2. **(C)** Zoomed-in view of the transmembrane domain of ADAM17 (color-coded by conservation) and TM1 of iRhom2 (gray) at Site 3. Annotated ADAM17 amino acids have side chains depicted as sticks. **(D)** Western blot analysis of ConA-enriched lysates transfected with the ADAM17 mutations used in Figures 4B-C. The zymogen and mature forms of ADAM17 are marked with arrows.

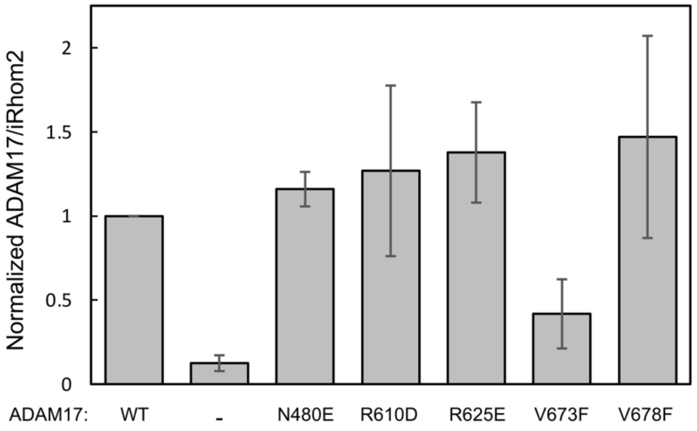

Supplementary Figure 6: Quantification of the Co-immunoprecipitation Between ADAM17 Mutants and iRhom2. Densitometric analysis was performed on western blots from the immunoprecipitation (IP) experiments in Figure 4B, assessing the interaction between mVenus-iRhom2 and ADAM17 mutants. Bars represent the average of the normalized ratio of ADAM17 (detected with αADAM17) to iRhom2 (detected with αGFP) in each immunoprecipitated sample. Error bars represent the mean ± SD from $N = 3$ independent experiments.

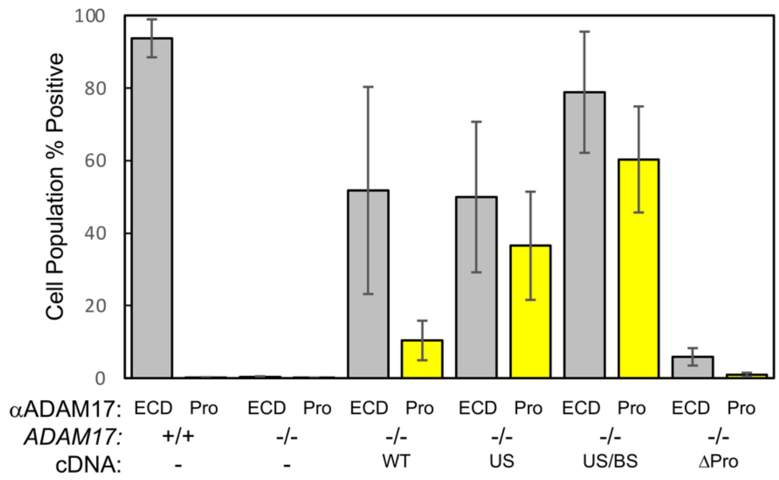

Supplementary Figure 7: Quantification of ADAM17 and the Prodomain on the Cell Surface. Quantification of flow cytometry analysis of the surface amount of ADAM17 (grey bars) and Prodomain (yellow bars) on the cell surface of WT and *ADAM17*$^{-/-}$ U2OS cells transfected with ADAM17 expression cDNA constructs used in Figure 5. Error bars represent the mean ± SD from *N = 3* independent experiments.

REFERENCES

1. Lipper CH, Egan ED, Gabriel KH, Blacklow SC. Structural basis for membrane-proximal proteolysis of substrates by ADAM10. *Cell* **186**, 3632-3641 e3610 (2023).

2. Seegar TCM*, et al.* Structural Basis for Regulated Proteolysis by the alpha-Secretase ADAM10. *Cell* **171**, 1638-1648 e1637 (2017).

3. Liu C, Xu P, Lamouille S, Xu J, Derynck R. TACE-mediated ectodomain shedding of the type I TGF-beta receptor downregulates TGF-beta signaling. *Mol Cell* **35**, 26-36 (2009).

4. Goehring A*, et al.* Screening and large-scale expression of membrane proteins in mammalian cells for structural studies. *Nat Protoc* **9**, 2574-2585 (2014).

5. Weissmann F*, et al.* biGBac enables rapid gene assembly for the expression of large multisubunit protein complexes. *Proc Natl Acad Sci U S A* **113**, E2564-2569 (2016).

6. Koushik SV, Chen H, Thaler C, Puhl HL, 3rd, Vogel SS. Cerulean, Venus, and VenusY67C FRET reference standards. *Biophys J* **91**, L99-L101 (2006).

7. Punjani A, Rubinstein JL, Fleet DJ, Brubaker MA. cryoSPARC: algorithms for rapid unsupervised cryo-EM structure determination. *Nat Methods* **14**, 290-296 (2017).

8. Bepler T*, et al.* Positive-unlabeled convolutional neural networks for particle picking in cryo-electron micrographs. *Nat Methods* **16**, 1153-1160 (2019).

9. Tunyasuvunakool K*, et al.* Highly accurate protein structure prediction for the human proteome. *Nature* **596**, 590-596 (2021).

10. Pettersen EF*, et al.* UCSF Chimera--a visualization system for exploratory research and analysis. *J Comput Chem* **25**, 1605-1612 (2004).

11. Emsley P, Cowtan K. Coot: model-building tools for molecular graphics. *Acta Crystallogr D Biol Crystallogr* **60**, 2126-2132 (2004).

12. Afonine PV*, et al.* Towards automated crystallographic structure refinement with phenix.refine. *Acta Crystallogr D Biol Crystallogr* **68**, 352-367 (2012).

13. Sanchez-Garcia R, Gomez-Blanco J, Cuervo A, Carazo JM, Sorzano COS, Vargas J. DeepEMhancer: a deep learning solution for cryo-EM volume post-processing. *Commun Biol* **4**, 874 (2021).

14. Maretzky T*, et al.* iRhom2 controls the substrate selectivity of stimulated ADAM17-dependent ectodomain shedding. *Proc Natl Acad Sci U S A* **110**, 11433-11438 (2013).

15. Sahin U, Weskamp G, Zheng Y, Chesneau V, Horiuchi K, Blobel CP. A sensitive method to monitor ectodomain shedding of ligands of the epidermal growth factor receptor. In: *Epidermal Growth Factor: Methods and Protocols* (eds T.B. Patel, Bertics PJ). Humana Press Inc. (2006).

16. Schlöndorff J, Becherer JD, Blobel CP. Intracellular maturation and localization of the tumour necrosis factor alpha convertase (TACE). *Biochem J* **347 Pt 1**, 131-138 (2000).

17. Ashkenazy H*, et al.* ConSurf 2016: an improved methodology to estimate and visualize evolutionary conservation in macromolecules. *Nucleic Acids Res* **44**, W344-350 (2016).
